# MiR156-dependent and miR156-independent effects of the RNA-binding protein *At*GRP7 on vegetative phase change in Arabidopsis

**DOI:** 10.1186/s12870-026-10084-w

**Published:** 2026-10-17

**Authors:** Stephan Weber, Alexander Steffen, Tino Köster, Martin Lewinski, Dorothee Staiger

**Affiliations:** RNA Biology and Molecular Physiology, Faculty of Biology, Bielefeld University, Bielefeld, 33615 Germany

**Keywords:** Arabidopsis, iCLIP, miRNA156, Phase change, RNA-binding protein

## Abstract

**Background:**

The life cycle of higher plants consists of a vegetative and a reproductive phase. The vegetative phase is further divided into a juvenile and an adult phase. Precise timing of the vegetative phase change, the transition from juvenile to adult vegetative phase, is crucial for plant fitness. A master regulator of vegetative phase change is the highly-conserved microRNA miR156. RNA-binding proteins (RBPs) are central regulators of post-transcriptional gene regulation and developmental plasticity, yet for many RBPs their direct RNA targets and the developmental relevance of these interactions remain incompletely understood.

**Results:**

Here, we identify the glycine-rich RBP *At*GRP7 as a regulator of vegetative phase change in *Arabidopsis thaliana*. Plants overexpressing *At*GRP7 (*At*GRP7ox) exhibited a prolonged juvenile vegetative phase with a reduced leaf emergence rate. Nevertheless, they flowered earlier than wild-type plants. In contrast, the *grp7-1* loss-of-function mutant transitioned earlier to the adult vegetative phase without changes in leaf emergence rate but displayed delayed flowering. Additional loss of the paralog *At*GRP8 in *grp7-1 grp8-2* plants did not further accelerate phase change, indicating a predominant role of *At*GRP7 in this process. To systematically identify pri-miRNA targets of *At*GRP7, we applied an adapted iCLIP2 workflow and detected in vivo binding of *At*GRP7 to 44 pri-miRNAs, including *MIR156A* and *MIR156C*. *At*GRP7 altered the abundance of *MIR156A* and *MIR156C* precursors, and expression of a miR156 target mimic (MIM156) partially suppressed the prolonged juvenile phase of *At*GRP7ox plants, indicating that altered miR156 activity contributes to the observed developmental phenotype. Expression of miR156-responsive SPL transcription factors was affected only modestly, suggesting that the developmental effects of *At*GRP7 cannot be explained solely by regulation of the canonical miR156-SPL pathway.

**Conclusion:**

Together, our findings identified *At*GRP7 as a regulator of vegetative phase change and established an adapted iCLIP2 workflow for identifying *At*GRP7-bound pri-miRNAs in vivo. Our data indicate that altered miR156 activity contributes to the developmental phenotype of *At*GRP7ox, while additional post-transcriptional mechanisms are likely involved. These findings extend the established role of *At*GRP7 in flowering time regulation and reveal a novel function in coordinating vegetative and reproductive development.

**Supplementary Information:**

The online version contains supplementary material available at https://doi.org/10.1186/s12870-026-10084-w.

## Background

Higher plants undergo distinct phases during their development. The vegetative phase is characterized by leaf development and the accumulation of biomass while the reproductive phase is characterized by the formation of flowers and the onset of seed production. The vegetative phase can be further separated into a juvenile and an adult vegetative phase. Correct timing of the transition between juvenile and adult vegetative phase is essential for plant fitness and is therefore regulated by a multitude of genetically separable pathways, which in turn are modulated by both, environmental and endogenous cues [1–4].

In *Arabidopsis thaliana*, this transition from the juvenile to the adult vegetative phase is accompanied by alterations in several phenotypic traits. Whereas juvenile leaves have long petioles, a round shape with smooth leaf margins, and produce trichomes only on the adaxial (upper) side of the leaf blade, adult leaves have short petioles, narrow leaf blades with serrated margins and trichomes on both, the adaxial and abaxial (lower) leaf surface [5, 6].

The transition from the juvenile to the adult vegetative phase is a tightly regulated process. At the core of the regulation lies the microRNA miR156 and its targets [1, 3, 7–9]. MicroRNAs (miRNAs or miRs) are a class of small 20 to 24 nt long noncoding single-stranded RNAs. They regulate gene expression by base-pairing to cognate target mRNAs, leading either to cleavage of the mRNA or translational inhibition. miRs are encoded by *MIR* genes located either in intergenic regions or in intronic regions of protein-coding genes and other noncoding RNAs. In plants, *MIR* genes are transcribed by DNA-dependent RNA polymerase II. The resulting primary miRs (pri-miRs) are folded into hairpin-like structures consisting of a terminal loop, an upper stem, the miR/miR* region, a lower stem, and additional unstructured regions. The mature miR is processed from this precursor by two subsequent cleavage events catalyzed by the dicing complex, consisting of the RNase III endonuclease DICER-LIKE 1 (DCL1), the dsRNA-binding protein HYPONASTIC LEAVES 1 (HYL1), and the zinc finger protein SERRATE (SE). The resulting miRNA/miRNA* duplexes are then loaded into ARGONAUTE1 (AGO1). The passenger miRNA* strand is subsequently degraded, while the guide miRNA strand is retained in AGO1 to form the RNA-induced silencing complex (RISC, reviewed in [10, 11]).

In Arabidopsis, the highly conserved miR156 and the closely related miR157 are master regulators of the juvenile-to-adult phase transition [9, 12], whereby the effect of miR156 seems to be dominant [7, 13]. MiR156 is encoded by a multigene family comprised of eight loci (*MIR156A* to *MIR156H)*, with *MIR156A* and *MIR156C* being the major sources of mature miR156 [7]. Plants with an elevated level of miR156 show a markedly prolonged juvenile phase during which they produce a multitude of leaves with juvenile traits, characterized by smaller rounder leaves without serrated margins and no trichomes on the abaxial surface [8]. To study the effect of reduced levels of miR156, plants that express a mimicry construct (MIM156) that effectively sequesters mature miR156 from all *MIR156* loci can be used. These MIM156 plants show a severely shortened juvenile phase or no juvenile phase at all and consequently flower shortly after germination [8, 14, 15].

The miR156 negatively regulates 10 out of 16 *SQUAMOSA PROMOTER BINDING PROTEIN-LIKE (SPL)* transcription factors, constituting the aging pathway: namely, SPL2/3/4/5/6/9/10/11/13 and 15 [16–19]. SPLs and their target genes promote the development of adult vegetative traits, floral induction, and floral meristem identity as well as regulating processes involved in root and shoot development [7, 19–21]. The miR156-SPL module regulates vegetative phase change in a threshold-dependent manner. MiR156 is strongly expressed during early seedling stages, and its expression subsequently decreases with further development [3]. With decreasing miR156 levels, the repression of SPL transcription factors subsides, and an increase in SPLs activates their respective targets, leading to the juvenile-to-adult transition [7]. During early development, *SPL* expression in emerging leaves is repressed by shoot apical meristem (SAM)-produced miR156. As juvenile growth progresses, *SPL* expression in leaves is repressed mainly by leaf-produced miR156, while miR156 from the SAM plays only a minor role at these stages. The temporal decline in miR156 levels across the shoot apex eventually leads to *SPL* derepression and triggers vegetative phase change [22]. The main genes responsible for the vegetative phase change are *SPL9*/*13*/*15*, whereas *SPL3*/*4*/*5* additionally promote floral meristem identity transition [3, 7, 9, 16]. *SPL2*/*10* and *11* are expressed at low levels and are responsible for mediating adult resistance to *P. syringae* [3, 19, 23]. The function of *SPL6* is unknown but is associated with disease resistance [24].

The circadian clock–regulated RNA-binding protein (RBP) *Arabidopsis thaliana* glycine-rich RNA-binding protein 7 (*At*GRP7) influences flowering time, where loss-of-function mutants flower late and overexpression accelerates flowering, particularly under short days (SDs) [25–27]. *At*GRP7 also affects pri-miR processing for a suite of miRs, with overexpression causing pri-miR accumulation and reduced mature miR levels [28]. At the molecular level, *At*GRP7 modulates alternative splicing, RNA stability, and autoregulates itself and its paralogue *At*GRP8 through alternative splicing coupled with nonsense-mediated decay [29–36]. In addition, *At*GRP7 contributes to immunity and abiotic stress resistance [35, 37–40].

To determine the RNA interaction partners of RBPs globally, *crosslinking and immunoprecipitation* (CLIP) techniques [41–43] have been established. *Individual nucleotide resolution crosslinking and immunoprecipitation* (iCLIP) uses UV light-mediated crosslinking to form covalent bonds between RNA and bound proteins, and the termination of the reverse transcription reaction at the crosslinked nucleotide allows for the identification of binding sites after sequencing. Thus, binding motifs can be determined with nucleotide resolution. Using plants expressing *At*GRP7 fused to GREEN FLUORESCENT PROTEIN (GFP) under control of the native promoter in *grp7-1* (*AtGRP7::AtGRP7-GFP grp7-1*), iCLIP identified 2708 *At*GRP7 target transcripts [30, 31, 44]. Recently, the iCLIP protocol was refined and the optimized iCLIP2 enables efficient, high-resolution mapping of in vivo RNA–protein interactions in plants through more stringent purification and streamlined library preparation [44].

Owing to the low crosslinking efficiency of UV light, iCLIP is biased towards more abundant transcripts, which previously prevented the reliable detection of pri-miR transcripts bound by *At*GRP7. To detect low-abundance pri-miRs, we introduced *AtGRP7::AtGRP7-GFP* into the *hyl1-2* mutant that accumulates pri-miRs due to a deficiency in the microprocessor complex component HYL1 [45, 46].

Here, we investigated the role of *At*GRP7 in vegetative development and its potential impact on the juvenile-to-adult phase transition. To identify pri-miRs involved in developmental regulation that are bound by *At*GRP7, we applied iCLIP2 [44] in the *hyl1-2* background. We focused on miR156, a key regulator of developmental timing, and examined how *At*GRP7 affects miR156 levels and the expression of miR156-targeted SPL transcription factors. To study the effect of reduced levels of miR156 we made use of a target mimicry against miR156, sequestering miR156A-miR156H. Finally, we assessed how these interactions shape vegetative phase transitions and developmental plasticity. Taken together, the results of this study provide insights into how *At*GRP7 contributes to the regulation of vegetative phase change in plants.

## Methods

### Plant material

The *At*GRP7 T-DNA line, *grp7-1*, carries a T-DNA insertion in the 5´UTR [47, 48]. The *atgrp7 atgrp8* double mutant *grp7-1 grp8-2* was generated by crossing *grp7-1* with the *grp8-2* CRISPR/Cas9 mutant harboring a 29 bp deletion 16 nucleotides downstream of the AUG start codon [30, 49]. *AtGRP7*-overexpressing transgenic lines were generated by ectopically overexpressing *AtGRP7* under the control of the Cauliflower Mosaic Virus (CaMV) 35 S promoter with a duplicated enhancer in the Col-2 ecotype [29, 50]. The target mimicry line against miR156, MIM156, has been described [14, 15]. MIM156 plants were crossed with *grp7-1*, *grp7-1 grp8-2*, and *At*GRP7ox. Backcrosses of *grp7-1* MIM156 plants were generated by crossing homozygous plants once again with MIM156 plants. Experiments were performed with homozygous F_3_ plants. The plants used for iCLIP2 were generated by crossing *AtGRP7::AtGRP7-GFP grp7-1* [31] with the *hyl1-2* mutant. The resulting crosses were genotyped and homozygous F_3_ lines were used for the experiments.

### Determination of the leaf emergence rate

Seeds were sown on soil, stratified for 2 d at 4 °C, germinated and grown under SD (8 h light / 16 h dark) conditions. The plants were grown in a Percival Incubator SE59-AR3cLED (CLF Plant Climatics, Wertingen, Germany) at 20 °C. The number of visible rosette leaves was counted three times a week until plants flowered. The mean values ± standard deviations were calculated.

### Determination of juvenile-to-adult vegetative phase change

Seeds were sown on soil, stratified for 2 d at 4 °C, germinated and grown under SD conditions. Plants were grown in a Percival Incubator SE59-AR3cLED (CLF Plant Climatics, Wertingen, Germany) at 20 °C. The first leaf with abaxial trichomes was determined by counting all leaves without abaxial trichomes using a binocular. The mean values ± standard deviations were calculated, and statistical significance was tested by one-way ANOVA with post-hoc Tukey´s HSD (Additional File 6). For leaf shape analysis, seeds were sown on soil, stratified for 2 d at 4 °C and germinated and grown under SD conditions at 20 °C. Fully expanded leaves from 21 to 25 plants were cut, attached to paper from oldest to youngest leaf using double-sided tape, flattened with clear tape and then scanned. After scanning, angles of the base of leaf 11 were measured for each plant using Fiji [51]. Length-to-width ratios were determined using the scanned leaves with Fiji by measuring the longest and the widest distance of leaf 11 for each plant. For both metrics, statistical significance was tested by one-way ANOVA with post-hoc Tukey´s HSD.

### Quantitative real-time RT-PCR

Quantitative real-time RT-PCR (qRT-PCR) was performed as previously described [34]. The primers used are listed in Additional File 7. Analysis of *SPL3* in *At*GRP7ox MIM156 and *grp7-1* MIM156 was performed using 6-week-old plant material grown under SD conditions. For the detection of mature miR156, a TaqMan^®^ stem-loop RT-qPCR assay (ThermoFisher Scientific, Catalog # 4427975, Assay-ID 000333) was used. SnoR101 (ThermoFisher Scientific, Catalog # 4427975, Assay-ID 001698) served as reference.

### Plant iCLIP2

Plant iCLIP2 was performed on total lysates from 14 d old *AtGRP7::AtGRP7-GFP grp7-1 hyl1-2* seedlings grown in 12 h L / 12 h D cycles at 20 °C according to the iCLIP2 protocol [44]. Briefly, RNA–protein complexes were crosslinked using 254 nm UV-light at a dose of 2000 mJ/cm^2^ and subsequently recovered from crude extracts by immunoprecipitation with GFP Trap beads (Proteintech, Planegg-Martinsried, Germany). The RNA-protein complexes were radiolabeled, resolved on an SDS-PAGE gel and blotted onto a nitrocellulose membrane. RNA was released from the membrane by performing a proteinase K treatment, (Additional File 1, Fig. S3A; Additional File 2: Uncropped autoradiogram to Fig. S3A). The purified RNA was reverse transcribed to cDNA and subsequently processed according to the iCLIP2 library preparation protocol [44]. Sequencing reads were analyzed using our established bioinformatics workflow [44]. To assess unspecific binding via the GFP moiety, iCLIP2 crosslink tracks of plants expressing GFP-only under control of the CaMV 35 S promoter (35 S::GFP) were used from [52].

### *MIR* annotation file

The annotations of the genomic pri-miR coordinates from mirEX2 [53] were combined with data from Lepe-Soltero et al. [54]. Annotations with the longest genomic range for each miR were transformed into BED and GTF formats. The sources, from which the annotations originate were specified in the naming field of the file, i.e., miRXXX_M2 for mirEX2 or miRXXX_LS for Lepe-Soltero. Pri-miRs with no counterpart in the other source were marked with oM2 (only miREX2) or oLS (only Lepe-Soltero).

## Results

### *At*GRP7 affects vegetative development

The transition from vegetative to reproductive growth is influenced by *At*GRP7, as the *grp7-1* loss-of-function mutant has a late-flowering phenotype and overexpression of *At*GRP7 leads to early flowering [25, 26]. Given that *At*GRP7 is involved in flowering time, we investigated whether *At*GRP7 also influences the other major developmental transition, the vegetative phase change.

**Fig. 1:**
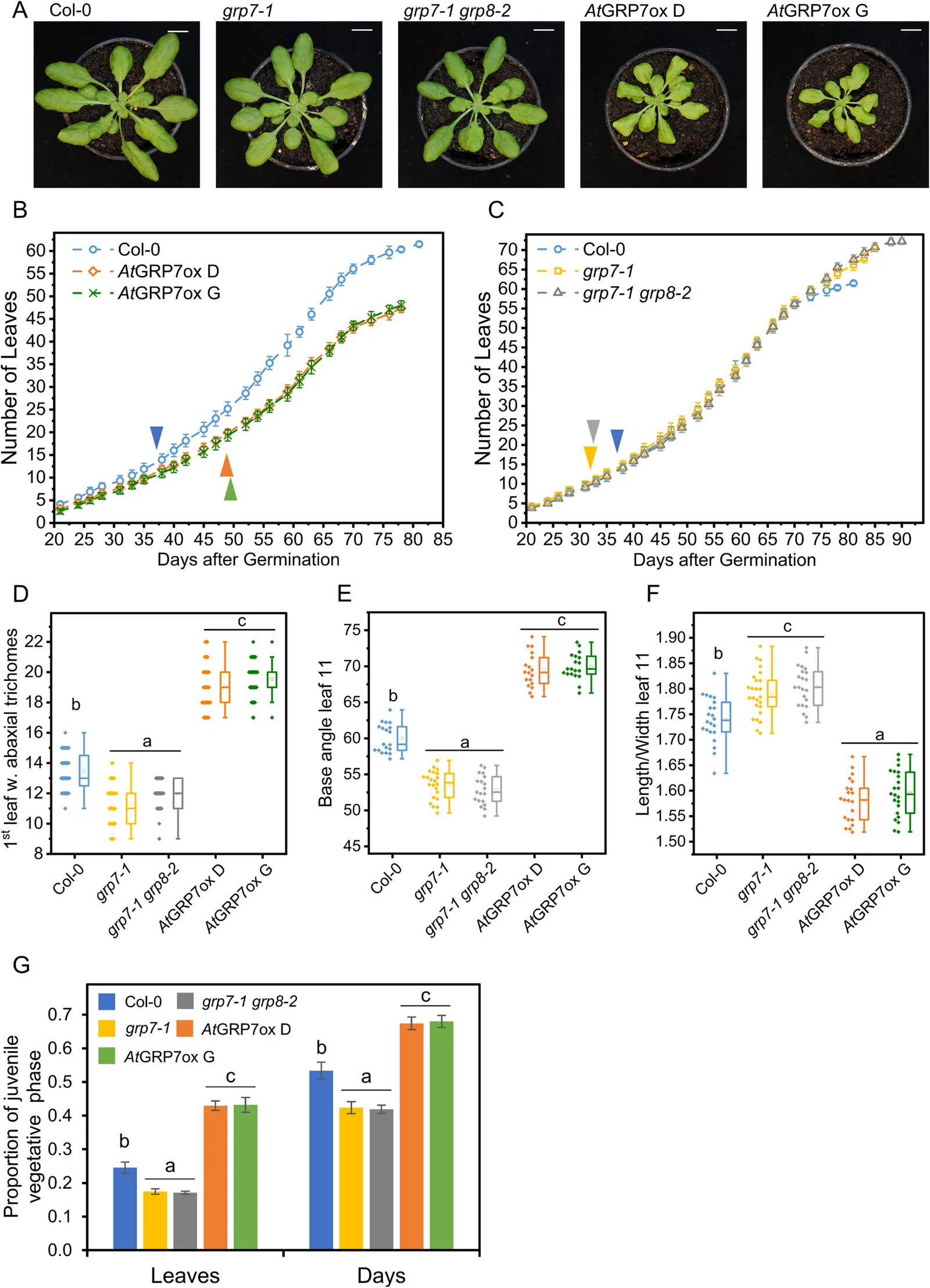
*At*GRP7ox promotes a prolonged juvenile vegetative phase. **A** Representative phenotype of 5-week-old Col-0, *grp*7-1, *grp*7-1 *grp*8-2, *At*GRP7ox D and *At*GRP7ox G plants. The white bar scales to 1 cm. **B** Leaf emergence of Col-0 and two independent *At*GRP7ox lines, and (**C**) of Col-0, *grp*7-1, and *grp*7-1 *grp*8-2 (*n* = 25). All visible leaves were counted three times per week until flowering. Data are shown as mean ± SD. The first appearance of abaxial trichomes is indicated by triangles. **D** Leaf number at the appearance of the first abaxial trichome in Col-0, *grp*7-1, *grp*7-1 *grp*8-2, and two *At*GRP7ox lines (*n* = 48). **E** Base angle of leaf 11 and (**F**) length-to-width ratio of leaf 11 in Col-0, *grp*7-1, *grp*7-1 *grp*8-2, and two *At*GRP7ox lines (*n* = 21–25). Data are shown as box-and-whisker plots (median, mean, 25th–75th percentiles, and 5th–95th percentiles) with individual data points overlaid. Different letters indicate significant differences (Tukey's HSD, *p* < 0.05). **G** Proportion of the juvenile vegetative phase until flowering, represented as the ratio of the first leaf with abaxial trichomes to the number of leaves at flowering or using days instead of leaves. Different letters indicate statistical significance at *p* < 0.05 (Tukey's HSD). All plants were grown in SD conditions

We determined the leaf emergence rates of plants constitutively overexpressing *At*GRP7 (*At*GRP7ox) [26, 29, 50] as well as those of the T-DNA insertion line *grp7-1* [47, 48] and the double loss-of-function line *grp7-1 grp8-2* [30, 49] grown under SD conditions by counting all visible leaves three times a week until the plants started flowering. Compared to wildtype (wt) Col-0, the two independent *At*GRP7ox lines exhibited a reduced leaf emergence rate throughout vegetative development, leading to the production of fewer leaves over time (Fig. 1A and B; Additional File 1, Fig. S1A). The divergence between wt and the *At*GRP7ox lines became particularly apparent from day 40 onwards. Furthermore, both *At*GRP7ox lines transitioned to reproductive growth earlier than wt Col-0 and therefore stopped producing new leaves approximately three days earlier. This is consistent with the previously described early flowering phenotype of *At*GRP7ox plants [25, 26]. Overexpression of *AtGRP7* also lead to smaller rosette sizes and altered leaf morphology compared to wt (Fig. 1A; Additional File 1, Fig. S1C). The leaf emergence rates of *grp7-1* and *grp7-1 grp8-2* were similar to that of wt Col-0 (Fig. 1A and C; Additional File 1, Fig. S1B). Rosette size and leaf morphology did not differ between wt and the knockout lines (Fig. 1A; Additional File 1, Fig. S1C). Differences in leaf emergence were only visible from day 75 onward when Col-0 plants started to flower and stopped the vegetative growth while the loss-of-function lines still developed more leaves. Additional loss of *At*GRP8 in the *grp7-1 grp8-2* loss-of-function line did not alter the leaf emergence rate but further delayed flowering. This is in accordance with the reported late-flowering phenotype of *grp7-1* and *grp7-1 8i*, plants with RNA interference-mediated reduction of *At*GRP8 [25–27]. Thus, *AtGRP7* overexpression slowed vegetative growth by reducing the rate of leaf emergence while promoting early flowering, whereas loss of *AtGRP7* or *AtGRP7*/*AtGRP8* did not affect leaf emergence rate but delayed flowering.

Vegetative development can be divided into a juvenile and an adult vegetative phase. A commonly used trait to assess the juvenile-to-adult phase transition is the appearance of trichomes on the abaxial leaf surface in adult leaves [6]. Therefore, we used the appearance of the first abaxial trichome to determine the length of the juvenile phase in wt Col-0, *At*GRP7ox, *grp7-1* and *grp7-1 grp8-2*. The first abaxial trichome appeared in wt plants on average around leaf 13–14 and in the two *At*GRP7ox lines significantly later around leaf 19–20, indicating a prolonged juvenile vegetative phase. In *grp7-1* and *grp7-1 grp8-2* plants, abaxial trichomes appeared on average around leaves 11–12 and thus significantly earlier than they did in wt plants (Fig. 1D; Additional File 1, Fig. S1D and E, Table 1). These findings indicate that *At*GRP7 is important for regulating vegetative phase change under SD conditions. *At*GRP7ox prolonged the juvenile vegetative phase, whereas *grp7-1* and *grp7-1 grp8-2* accelerated the juvenile phase. The difference in trichome formation between *grp7-1* and *grp7-1 grp8-2* was not significant, suggesting that an additional effect from the loss of *At*GRP8 in the double mutant is negligible.

**Table 1:** Overexpression of *At*GRP7 leads to a delayed formation of abaxial trichomes

Genotype	1st leaf with abaxial trichomes (mean ± SD)	*p* value
Col-0	13.48 ± 1.19	
*grp7-1*	11.17 ± 1.24	0.000039
*grp7-1 grp8-2*	11.98 ± 0.92	0.004727
*At*GRP7ox D	19.04 ± 1.51	0.000000
*At*GRP7ox G	19.54 ± 1.14	0.000000

The appearance of the first abaxial trichome was measured under SD conditions (*n* = 48). Statistical significance was assessed via Kruskal Wallis test followed by post-hoc Dunn´s test against Col-0

During vegetative phase change, leaves undergo additional changes in shape and structure. The base angle of leaves becomes smaller and the length-to-width ratio increases, when leave blades change from a rounder shape to a more elongated shape with serrated margins [6]. Therefore, we also determined the base angle as well as the length-to-width ratio of leaf 11 from SD grown plants (Fig. 1E). We chose leaf 11 as benchmark, as this leaf was the last one with juvenile traits before wt plants were undergoing vegetative phase change. Measurements revealed a base angle of 60° for leaf 11 of wt Col-0 plants, whereas *grp7-1* and *grp7-1 grp8-2* plants had a significantly narrower base angle with 53°. In contrast, the two *At*GRP7ox lines had a wider base angle with 70°. This confirms the delayed vegetative phase transition in *At*GRP7ox and the accelerated vegetative phase transition in *grp7-1* and *grp7-1 grp8-2*.

The length-to-width ratio served as a third marker for vegetative phase change and was also determined for leaf 11 (Fig. 1F). While wt Col-0 plants had a length-to-width ratio of 1.74, *grp7-1* and *grp7-1 grp8-2* had a significantly higher ratio of 1.79 and 1.80, respectively. Overexpression of *AtGRP7* lead to a significantly reduced length-to-width ratio of 1.59, further strengthening the observations that *At*GRP7 alters the timing of vegetative phase transition under SD conditions.

Taken together, the appearance of the first abaxial trichome, the base angle as well as the length-to-width ratio of leaf 11 revealed that loss of *At*GRP7 accelerated the vegetative phase change. *Grp7-1* and *grp7-1 grp8-2* produced the first abaxial trichome on average two leaves and three to four days earlier than wt Col-0 (Fig. 1C) while also having a smaller leaf base angle as well as a higher length-to-width ratio on leaf 11 (Fig. 1E and F). Conversely, *At*GRP7ox prolonged the juvenile vegetative phase, with abaxial trichomes forming on average five to six leaves and ten days later than wt (Fig. 1B). *At*GRP7ox plants also showed a wider base angle and a smaller length-to-width ratio of leaf 11 compared to wt (Fig. 1E and F).

To quantify the relative duration of the juvenile vegetative phase across genotypes, we calculated the proportion of the juvenile phase relative to the total vegetative period using the two developmental metrics of leaf number and days until bolting. These two measures allow assessment of developmental timing independent of potential differences in the absolute growth rate.

We found that wt plants remained in the juvenile phase for approximately 25% of their total vegetative growth. In *grp7-1* and *grp7-1 grp8-2* plants, this was markedly reduced to approximately 17%, indicating a noticeably accelerated progression into the adult vegetative phase. In contrast, *At*GRP7ox plants presented a substantial extension of the juvenile period by devoting 43% of the entire vegetative phase to the juvenile phase (Fig. 1G).

The same pattern emerged when chronological rather than morphological parameters were examined. When considering days instead of leaf number, wt plants spent 53% of the time of the vegetative phase in the juvenile phase, whereas in the loss-of-function lines, the juvenile phase accounted for 42% of the total vegetative phase. *At*GRP7ox plants again presented a prolonged juvenile stage, with the juvenile phase accounting for 67% of the total duration of the vegetative phase (Fig. 1G). Both developmental measures consistently revealed the same trend. Overexpression of *At*GRP7 extended the juvenile vegetative phase, while loss of *At*GRP7 shortened it.

Taken together, these findings indicate that *At*GRP7 delays the juvenile-to-adult transition and reduces the leaf emergence rate but promotes flowering according to earlier observations. This indicates that the role of *At*GRP7 in developmental phase change is different from its role in flowering time regulation.

### Plant iCLIP2 reveals in vivo binding of *At*GRP7 to pri-miRs

As overexpression of *At*GRP7 leads to a prolonged juvenile vegetative phase, *At*GRP7 binds to pri-miRs in vivo, and several pri-miRs accumulate at the expense of the mature miR [28], we set out to investigate whether *At*GRP7 may interact with miR156 precursor transcripts as well as other pri-miRs.

To assess *At*GRP7 binding to pri-miRs, we first re-analyzed our previously published iCLIP1 dataset [31] for bound pri-miRs. However, this approach yielded only limited and inconclusive evidence for *At*GRP7 binding, as we found crosslink events at several *MIR* loci, but binding sites were called at only nine *MIR* genes. Of these nine, seven showed unambiguous *At*GRP7 binding without overlap of other protein-coding or noncoding transcripts. Binding sites to pri-miRs overlapping with other genes were excluded from further analysis, as overlapping annotations make it impossible to determine which transcript is ultimately bound (Additional File 1, Fig. S2A and B). To systematically capture in vivo* At*GRP7–pri-miR interactions, we applied the improved plant iCLIP2 protocol [44] using *AtGRP7::AtGRP7-GFP grp7-1 hyl1-2* plants, which accumulate pri-miRs because of impaired processing [45, 46].

Performing iCLIP2, we generated on average 19 million reads per replicate, of which 91% passed quality control. Approximately 10 million deduplicated, uniquely mapped reads per replicate were retained for downstream analysis (Additional File 1, Fig. S3B; Additional File 3). Based on these data, we identified nearly 31,000 reproducible binding sites across approximately 5,500 transcripts (Additional Files 4 and 5). To identify bound pri-miRs, we used a custom pri-miR annotation file (see Methods).

Using this approach, 44 *MIR* loci with *At*GRP7 binding sites were identified (Table 2). When the most recent AtRTD3 transcriptome annotation was applied, 18 loci unambiguously mapped to *MIR*s (Additional File 1, Fig. S4A, B and C): *MIR156C*, *MIR157C*, *MIR158A*, *MIR159B*, *MIR160C*, *MIR166A*, *MIR167A*, *MIR167D*, *MIR168A*, *MIR171B*, *MIR171C*, *MIR172B*, *MIR393B*, *MIR398B*, *MIR398C*, *MIR408*, *MIR414* and *MIR4228*. In 23 cases, binding sites in *MIRs* overlapped with protein coding genes or noncoding genes on the same strand, making identification of the bound transcript impossible without further experimental validation (Table 2). For *MIR863*, *MIR2936* and *MIR5634*, classification was difficult as the identified binding sites were close to the annotated stem-loop, but the *MIR* genes also overlapped with other genes (Additional File 1, Fig. S4D).

**Table 2:** List of all *MIRs* bound by *At*GRP7 identified by iCLIP2

Bound MIRs		MIRs overlapping with another gene annotation	
*MIR*	Number of binding sites	*MIR*	Number of binding sites
*MIR156C*	9	*MIR777*	1
*MIR157C*	2	*MIR779*	4
*MIR158A*	1	*MIR823*	3
*MIR159B*	4	*MIR825*	3
*MIR160C*	1	*MIR850*	11
*MIR166A*	5	*MIR852*	3
*MIR167A*	5	*MIR853*	1
*MIR167D*	3	*MIR856*	15
*MIR168A*	6	*MIR860*	7
*MIR171B*	1	*MIR1888A*	1
*MIR171C*	1	*MIR2112*	1
*MIR172B*	6	*MIR4221*	1
*MIR393B*	1	*MIR5014A*	24
*MIR398B*	11	*MIR5014B*	1
*MIR398C*	2	*MIR5016*	1
*MIR408*	3	*MIR5026*	11
*MIR414*	1	*MIR5027*	3
*MIR4228*	7	*MIR5629*	1
		*MIR5642B*	2
		*MIR5664*	1
		*MIR8166*	7
		*MIR8170*	3
		*MIR8176*	2
		*MIR863*	11
		*MIR2936*	10
		*MIR5634*	1

*MIRs* with *At*GRP7 binding sites detected in *AtGRP7::AtGRP7-GFP grp7-1 hyl1-2* plants. All bound *MIRs* are categorized as *bound* (binding sites map unambiguously to the *MIR*) or *overlapping with another gene annotation* (binding sites map to a *MIR* overlapping with another gene annotation)

Next, we analyzed the sequence properties of the identified binding sites. Consistent with previous reports showing that *At*GRP7 preferentially binds a guanine followed by a uracil stretch and a cytosine [30, 31], we observed the same characteristic uracil-rich motif flanked by guanine and cytosine in the iCLIP2 data generated from *AtGRP7::AtGRP7-GFP grp7-1 hyl1-2* plants. The nucleotide composition of the binding sites was similar across all groups, including all binding sites, *MIR* binding sites, and binding sites associated with unambiguously bound *MIRs* (Additional File 1, Fig. S3C).

Compared with iCLIP1 [31], iCLIP2 yielded, on average, ten times more crosslink sites on pri-miRs, likely due to both the optimized protocol as well as the usage of the *hyl1-2* mutant. For several pri-miRs, including *MIR156A*, *MIR156C*, and *MIR159B* (Additional File 1, Fig. S5A-C), substantially more crosslinks and multiple binding sites were detected in iCLIP2, which were not identified previously. In addition, improved binding site calling and filtering enhanced specificity, as exemplified by *MIR398B*, where we could previously show a negative effect of *At*GRP7 on processing, leading to accumulation of the *MIR398B* at the expense of mature miR398b [28]. Previously present spurious binding sites in iCLIP1 at *MIR398B* were eliminated (Additional File 1, Fig. S5D). Overall, iCLIP2 enabled a more sensitive and robust detection of *At*GRP7 binding sites on pri-miRs, including transcripts with previously weak crosslink signals.

### Potential structural prerequisites of *At*GRP7 binding to pri-miRs

As pri-miRs form characteristic secondary structures necessary for proper processing, we aimed to elucidate whether *At*GRP7 binding occurs preferentially in regions with distinct conformational properties. We therefore mapped the *At*GRP7 iCLIP2 binding sites on the stem-loop secondary structure predicted by RNAFold [55] using RNAstructure editor [56]. Consistent with previous findings that *At*GRP7 preferentially interacts with single-stranded and less structured regions [57], binding sites on predicted stem-loops were located in regions that were less likely predicted to form secondary structures.

Several pri-miRs, including *MIR156C*, *MIR160C*, *MIR168A*, and *MIR4228*, showed *At*GRP7 binding at the predicted apical loop of the stem-loop (Fig. 3G and Additional File 1, Fig. S6A–E). These loci also presented additional binding sites either upstream or downstream of the hairpin, with *MIR156C*, *MIR168A*, and *MIR4228* showing multiple binding sites outside that region (Additional File 1, Fig. S4A). *MIR159B* displayed binding at the predicted hairpin between the apical loop and the miR/miR* region, along with upstream sites, whereas *MIR393B* contained a single site within the miR/miR* region (Additional File 1, Fig. S4B, Fig. S6D–E). In all cases, binding occurred in predicted single-stranded, less structured regions of the hairpin. We further examined the spatial relationship between *At*GRP7 binding sites located within miRNA hairpins and experimentally determined DCL1 cleavage sites [58]. In several cases, *At*GRP7 binding sites were located close to DCL1 cleavage sites, with *MIR156C*, *MIR160C*, *MIR168A* and *MIR159B* showing binding sites within 11–26 nt of at least one cleavage site, whereas the *MIR393B* binding site was located further downstream (Fig. 3G, Additional File 1, Fig. S6A-E). The binding sites of *At*GRP7 on the other *MIR* transcripts were distributed upstream and downstream of the potential hairpin. Six *MIRs* showed binding sites exclusively downstream, four exclusively upstream, and two displayed binding sites on both sides of the predicted hairpin structure (Additional File 1, Fig. S4C).

**Fig. 2:**
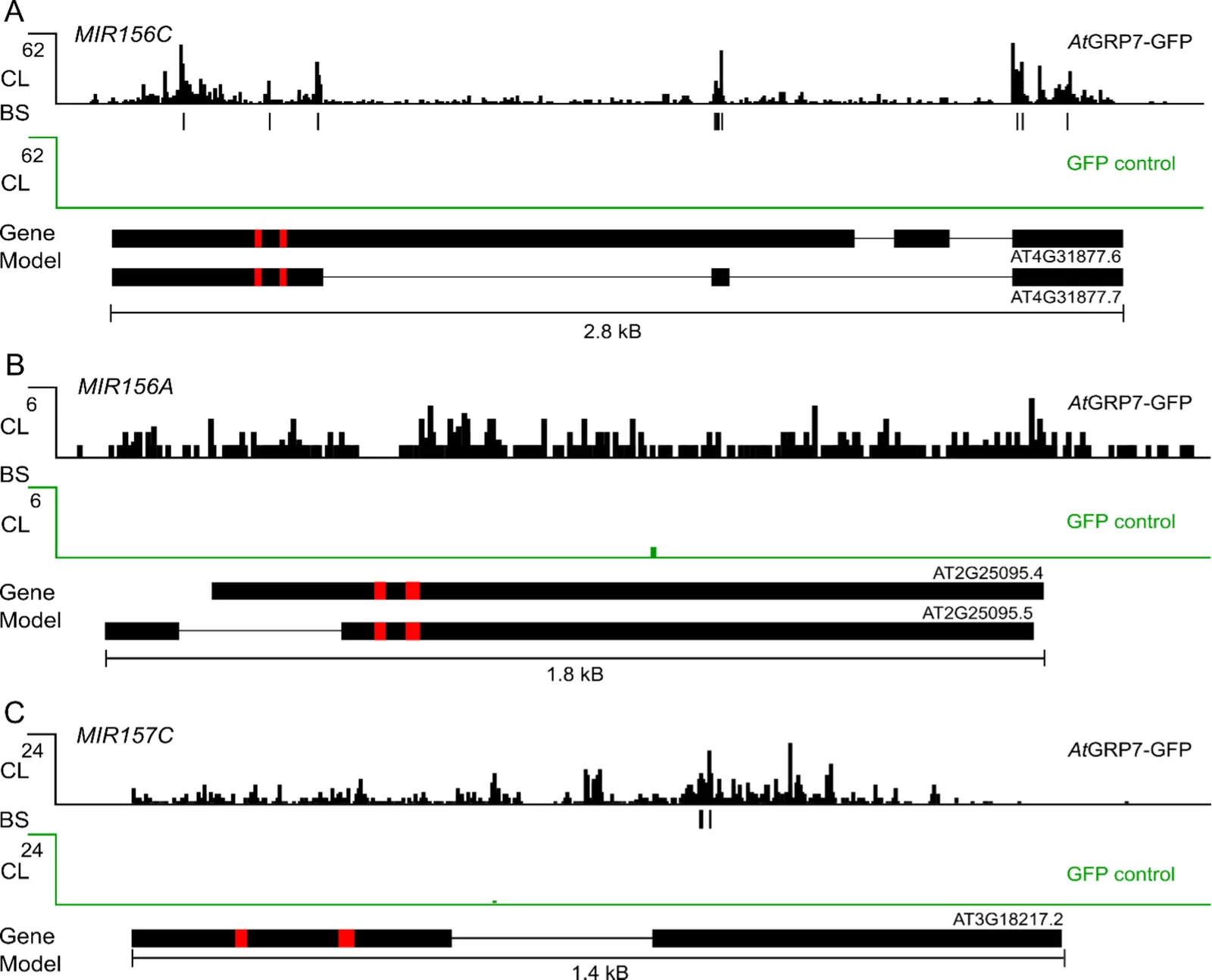
Detection of in vivo binding of *At*GRP7 to pri-miR156 through iCLIP2. *At*GRP7 iCLIP2 binding profiles of (**A**) *MIR156C*, (**B**) *MIR156A*, and (**C**) *MIR157C*. Crosslink (CL) tracks from the *AtGRP7::AtGRP7–GFP grp7-1 hyl1-2* sample (black) and the 35 S::GFP control (green) are shown. Called binding sites (BSs) are marked by black lines. Gene models of the most highly expressed pri-miR isoforms (based on RNA-seq data from [31]; annotated according to AtRTD3) are shown in 5´→3´ orientation, with exons (boxes), introns (lines), and the miR/miR* regions highlighted in red. The size of the displayed region is indicated

Among the bound *MIRs* were *MIR156C* and *MIR157C* (Fig. 2A, C), which are major precursors of *MIRs* regulating the vegetative phase change. Furthermore, crosslink events were also enriched at another major source of mature miR156, *MIR156A* (Fig. 2B), although no binding site was identified.

### *At*GRP7ox modulates *MIR156A* and *MIR156C* precursor abundance

As we observed changes in the length of the juvenile vegetative phase in plants with altered *At*GRP7 levels and observed in vivo binding of *At*GRP7 at *MIR156C* and *MIR156A*, the two dominant sources of mature miR156 [7], we aimed to further investigate the influence of *At*GRP7 on miR156. *At*GRP7ox, *grp7-1 grp8-2*, and wt Col-0 were grown under SD conditions and the levels of *MIR156A*, *MIR156C* and mature miR156 were monitored for four consecutive weeks at weekly intervals in the above-ground plant material.

**Fig. 3:**
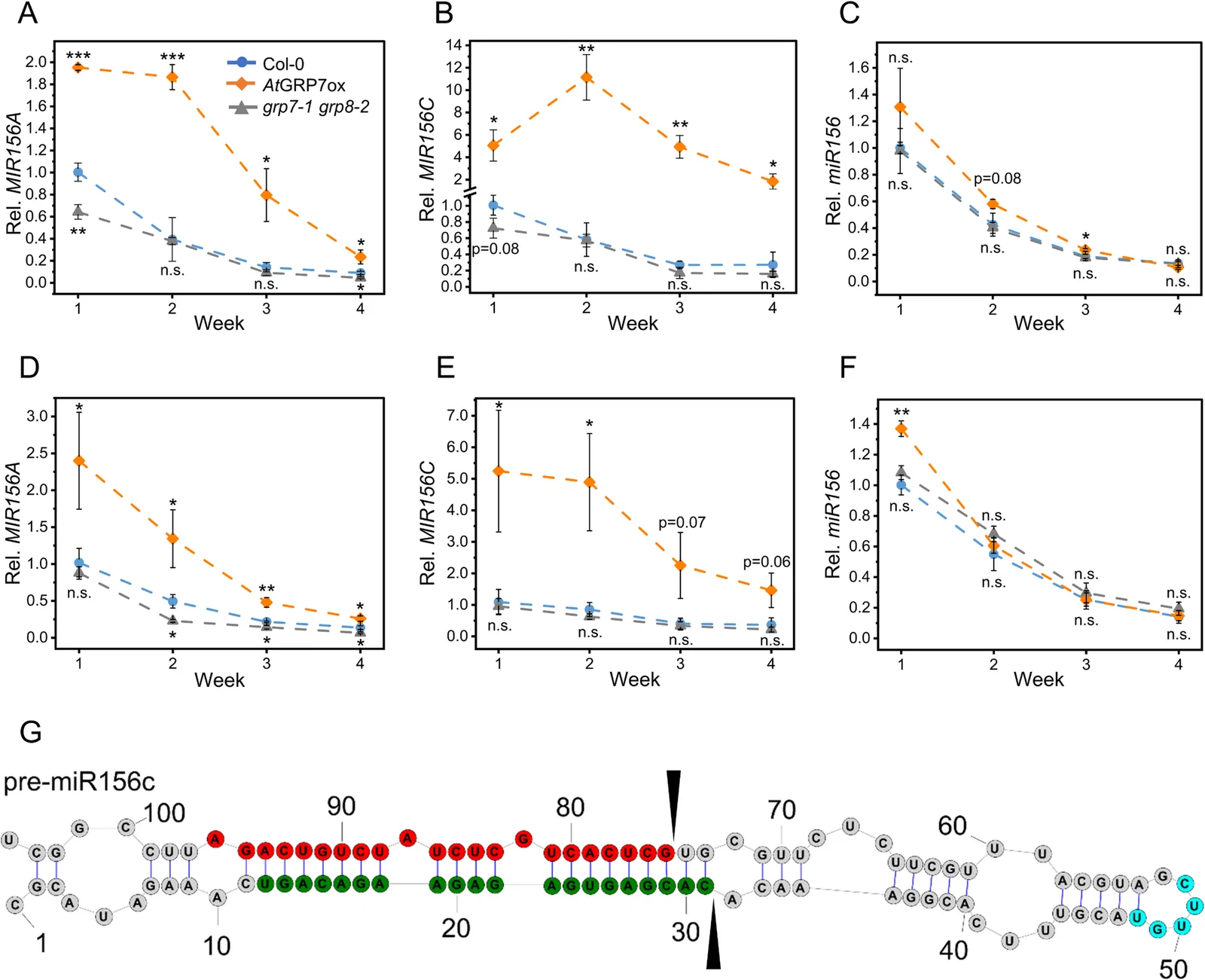
*At*GRP7ox affects the levels of *MIR156A* and *MIR156C. *Levels of (**A**,** D**) *MIR156A*, (**B**,** E**) *MIR156C*, and (**C**,** F**) mature *miR156* in Col-0, *grp7-1 grp8-2*, and *At*GRP7ox plants. Whole rosettes (**A**–**C**) or apically enriched tissue (**D**–**F**) from 1- to 4-week-old plants grown under SD conditions were analyzed via RT-qPCR. Expression values were calculated using the ΔΔCₜ method, normalized to *PP2A*, and set relative to Col-0 in week 1. Mature *miR156* was quantified by stem-loop RT-qPCR (TaqMan^®^), normalized to *snoR101*, and set relative to Col-0 in week 1. The data represent means ± SD of three biological replicates. Statistical significance compared with Col-0 was assessed by Student’s t-test (*** *p* ≤ 0.001; ** *p* ≤ 0.01; * *p* ≤ 0.05; n.s. *p* ≥ 0.05). **G** Secondary structure of pre-miR156c predicted by RNAFold. Highlighted are 5p-miR (green), 3p-miR (red) as well as *At*GRP7-GFP binding sites (cyan) determined by iCLIP2 in *AtGRP7::AtGRP7-GFP grp7-1 hyl1-2*. Experimentally determined DCL1 cleavage sites [58] are indicated by black arrows

The abundance of *MIR156A* was highest in the first week and then declined over time (Fig. 3A). Compared to wt, levels of *MIR156A* in *At*GRP7ox were increased significantly across all time points. In *grp7-1 grp8-2*, levels were significantly lower in week one. For *MIR156C,* the increase in *At*GRP7ox compared to wt was even greater. In contrast, *grp7-1 grp8*-*2* showed somewhat lower levels than wt, albeit not significantly. *MIR156C* was most abundant in week one in wt Col-0 and *grp7-1 grp8-2* and gradually decreased. This finding is in line with the proposed model where a reduction in miR156 levels enables the transition from the juvenile to the adult vegetative phase [3, 7]. Interestingly, in *At*GRP7ox, the abundance of *MIR156C* peaked in week two and then declined sharply (Fig. 3B), in line with the proposed mode of action.

Cell division in the SAM leads to the deposition of repressive histone marks on *MIR156* genes, thereby triggering a decrease in miR156 levels and allowing the subsequent vegetative phase change [59]. Therefore, *MIR156* levels were analyzed in apically enriched material, focusing on *MIR156* abundance in leaf primordia. Apically enriched material from wt Col-0, *grp7-1 grp8-2* and *At*GRP7ox plants was harvested once a week for four consecutive weeks. The levels of *MIR156A* were significantly elevated in *At*GRP7ox compared to wt Col-0 across all time points. Differences were especially apparent in weeks one and two. In contrast, *grp7-1 grp8-2* presented reduced levels of *MIR156A* across all time points (Fig. 3D). *MIR156C* was also significantly elevated in weeks one and two in *At*GRP7ox, and its level was lower in *grp7-1 grp8-2* than in wt plants, albeit not significantly (Fig. 3E).

Publicly available developmental expression data of *AtGRP7* from AtGenExpress, visualized using ePlant [60] showed strong expression in the shoot apex and developing vegetative tissues, while available data from mirEX2 [53] showed that *MIR156A* and *MIR156C* were also expressed in these tissues during early vegetative development (Additional File 1, Fig. S10A-C).

To test how differences in *MIR156A* and *MIR156C* were reflected in the level of mature miR156, we monitored miR156 by stem-loop RT-qPCR. The level of mature miR156 appeared higher in *At*GRP7ox throughout weeks one to three, whereas in *grp7-1 grp8-2*, no changes to wt Col-0 were detected (Fig. 3C). In the apically enriched material, miR156 significantly accumulated in *At*GRP7ox compared to wt Col-0 in week one. Again, there was no difference in the miR156 levels between wt Col-0 and *grp7-1 grp8-2* (Fig. 3F).

Together, the results of both experiments showed a strong elevation of levels of *MIR156A* and *MIR156C* in *At*GRP7ox compared with those in wt Col-0 under SD conditions, whereas they were reduced in *grp7-1 grp8-2*. Both precursors declined over time, matching the proposed mode of action of miR156. However, only in the case of *At*GRP7ox did the differences in pri-miR levels result in altered miR156 abundance at early developmental stages.

Besides miR156, the closely related miR157 has also been implicated in the regulation of vegetative phase change by targeting a largely overlapping set of SPL transcription factors [7, 13]. Therefore, we further investigated the influence of *At*GRP7 on miR157 as the most abundant precursor, *MIR157C*, was bound by *At*GRP7 (Fig. 2C). We additionally observed crosslinking events on *MIR157A*, another main precursor of miR157 (Additional File 1, Fig. S7A). Monitoring the levels of *MIR157A* and *MIR157C*, we observed a mild effect on the abundance of *MIR157A*. Levels were elevated in *At*GRP7ox starting from week 2 in both the total plant material and the apically enriched material (Additional File 1, Fig. S7B and D). For *MIR157C*, levels were reduced in total plant material in week one in *At*GRP7ox but otherwise not changed (Additional File 1, Fig. S7C). In the apically enriched material we observed no difference (Additional File 1, Fig. S7E). In *grp7-1 grp8-2*, we did not observe any effect on either precursor. Overall, the comparatively small effects on *MIR157* precursor abundance, together with the more pronounced effects on *MIR156* and the established predominant role of miR156 in vegetative phase transition [7], suggest that miR157 is unlikely to make a major contribution to the developmental phenotypes observed in plants with altered *At*GRP7 expression.

### Influence of *At*GRP7 on levels of SPL transcription factors

SPL transcription factors, which are involved in the regulation of vegetative development, are important downstream targets of miR156. Therefore, we monitored the expression of the key members responsible for the vegetative phase change, *SPL3*, *SPL9*, and *SPL15* in week 3 and week 4.

**Fig. 4:**
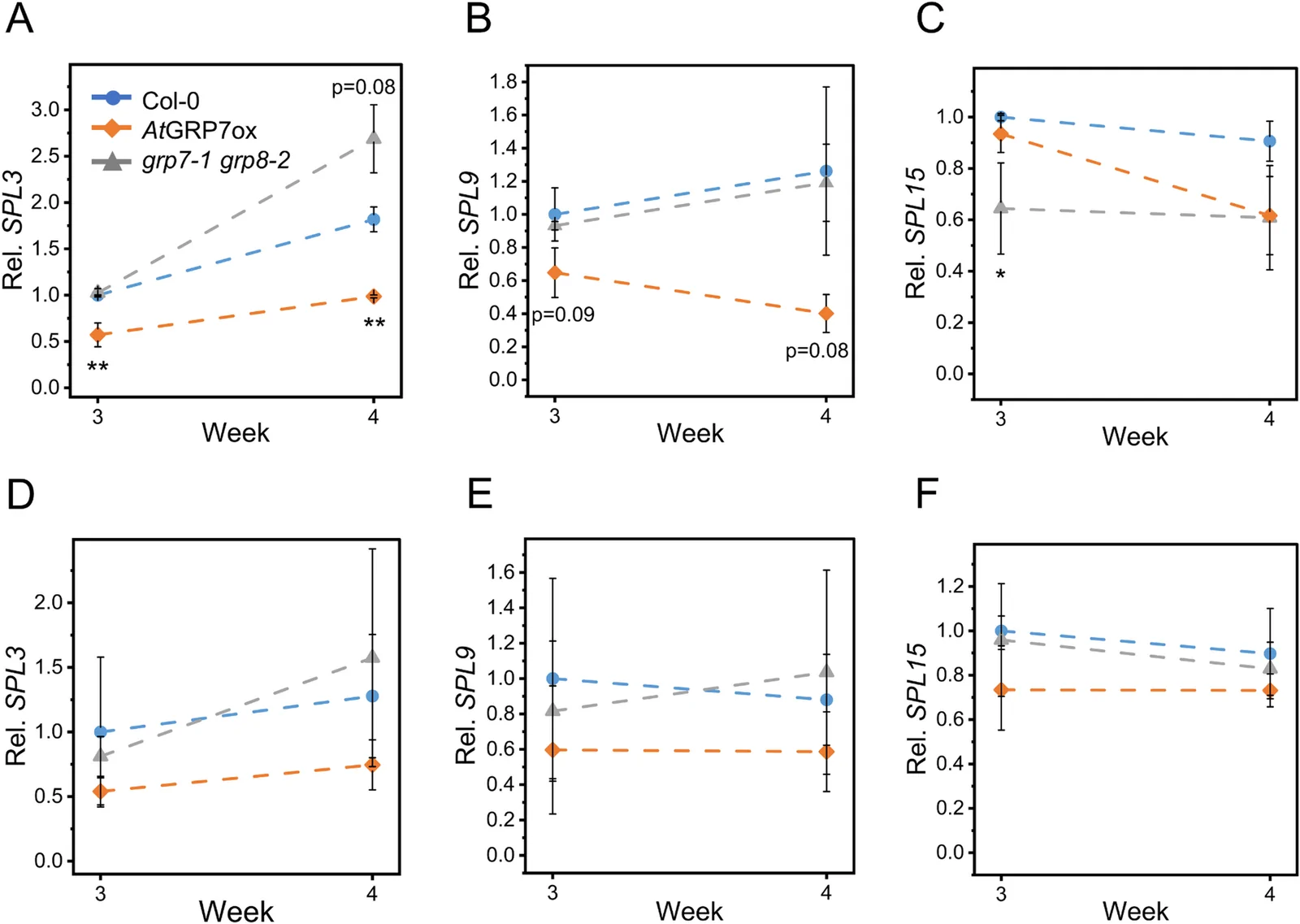
Influence of *At*GRP7 on *SPL* transcription factors. Levels of (**A**,** D**) *SPL3*, (**B**,** E**) *SPL9* and (**C**,** F**) *SPL15* in Col-0, *grp7-1 grp8-2*, and *At*GRP7ox plants. Whole rosettes (**A**–**C**) or apically enriched tissue (**D**–**F**) from 3- to 4-week-old plants grown under SD conditions was analyzed by RT-qPCR. The expression values were calculated using the ΔΔCₜ method, normalized to *PP2A*, and set relative to Col-0 at week 3. The data represent means ± SD of three biological replicates. Statistical significance compared to Col-0 was assessed by Student’s t-test (** *p* ≤ 0.01; * *p* ≤ 0.05), only significant differences were indicated

We found that *SPL3* levels in *At*GRP7ox plants remained significantly lower at both time points, whereas in *grp7-1 grp8-2* plants, *SPL3* levels were elevated at week 4 compared to those in wt plants (Fig. 4A). *SPL9* remained lower in *At*GRP7ox plants but showed no clear difference between *grp7-1 grp8-2* and wt plants (Fig. 4B). For *SPL15*, no differences in expression were observed in any genotype, except lower levels in week three for *grp7-1 grp8-2* compared to wt (Fig. 4C).

In the apically enriched material, levels of *SPL3* were lower in *At*GRP7ox as well, whereas they were slightly increased in *grp7-1 grp8-2* in week four, albeit not significantly (Fig. 4D). The levels of *SPL9* were affected in a similar manner. They were lower in *At*GRP7ox but were not markedly different between Col-0 and *grp7-1 grp8-2* (Fig. 4E). Levels of *SPL15* were slightly lower in *At*GRP7ox, albeit not significantly and we observed no differences in expression between *grp7-1 grp8-2* and wt (Fig. 4F).

Taken together, *At*GRP7ox had a mild effect on *SPL* expression levels, whereas they remained largely unchanged in *grp7-1 grp8-2* plants. *SPLs* relevant for vegetative phase change, *SPL9* and *15*, were lower in *At*GRP7ox but not significantly, indicating that *At*GRP7 might influence vegetative phase transition through SPL-independent mechanisms downstream or in parallel to miR156.

### Effect of miR156 target mimicry on the phenotype of *At*GRP7 mutants

As we observed a prolonged juvenile vegetative phase along with increased levels of *MIR156A* and *MIR156C* in *At*GRP7ox plants, we investigated whether this delayed vegetative phase change was dependent on miR156. Therefore, we introgressed a target mimicry construct against miR156 (MIM156) [14] into *At*GRP7ox, *grp7-1* and *grp7-1 grp8-2* to simultaneously inhibit the action of miR156 stemming from all *MIR156* precursors of the multi-gene family.

**Fig. 5:**
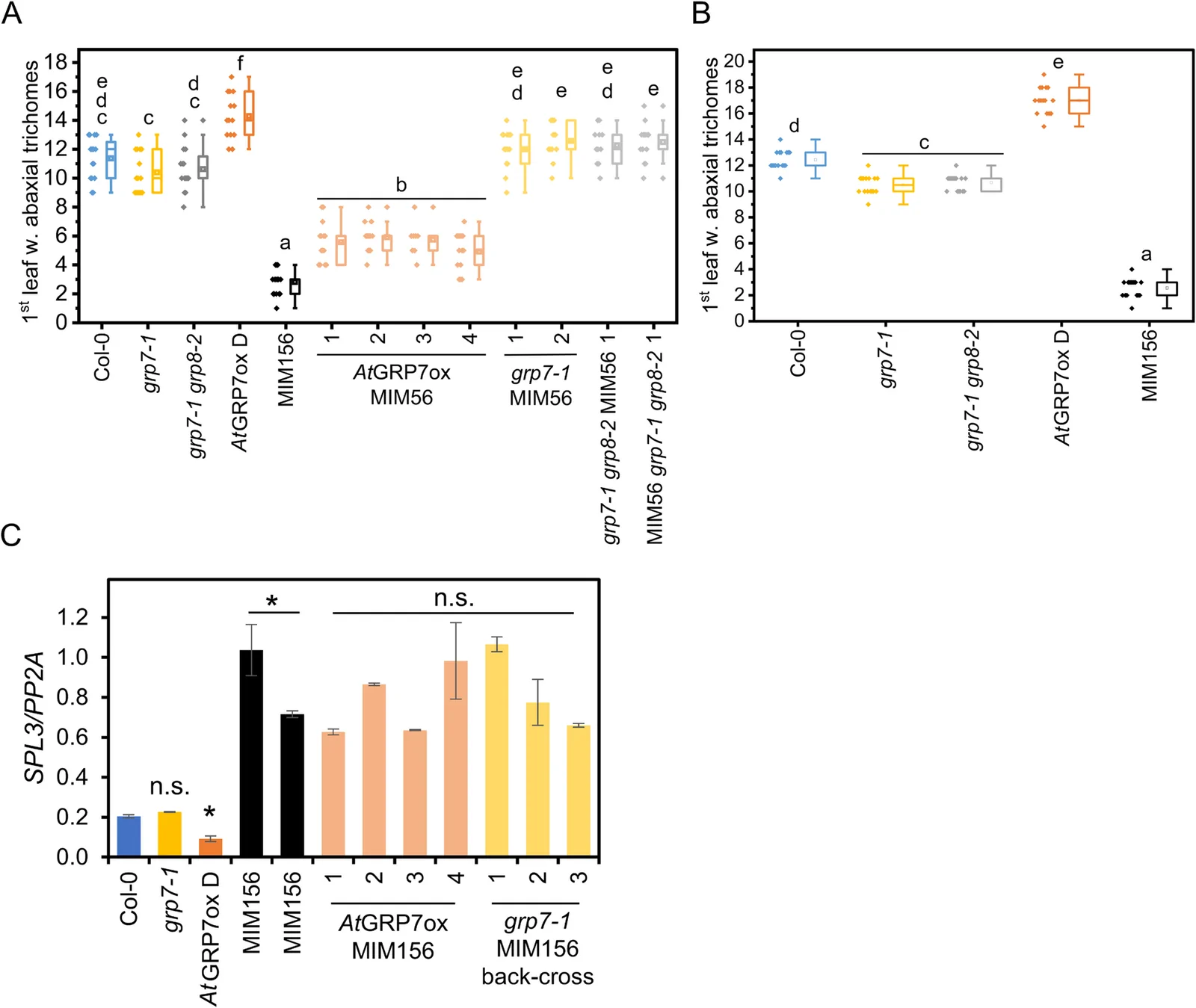
Effects of miR156 target mimicry on the phenotype of *At*GRP7 mutants. **A** Leaf number at the appearance of the first abaxial trichome in Col-0, *grp*7-1, *grp*7-1 *grp*8-2, *At*GRP7ox, MIM156, and the respective crosses (n = 17) grown under SD conditions. **B** A representative boxplot is shown, with median, mean, 25th–75th percentiles, and 5th–95th percentiles. Individual data points are overlaid. Cutout from Additional File 1, Fig. S8E. Different letters indicate significant differences (Tukey’s HSD, p < 0.05). **C** SPL3 expression in 6-week-old Col-0, *grp*7-1, *At*GRP7ox, MIM156, *At*GRP7ox MIM156, and *grp*7-1 MIM156 plants (RT-qPCR, normalized to *PP2A*; mean ± SD). Statistical significance (Student’s t-test) is indicated: *grp*7-1, *At*GRP7ox, MIM156 vs. Col-0; *At*GRP7ox MIM156, *grp*7-1 MIM156 vs. MIM156 (*** *p* ≤ 0.001; ** *p* ≤ 0.01; * *p* ≤ 0.05; n.s. *p* ≥ 0.05)

We then analyzed abaxial trichome formation under SDs in homozygous F_3_ plants and in the parental lines. In line with reported findings [14, 15], MIM156 plants presented a drastically shortened juvenile phase, with the first abaxial trichome appearing as early as leaf two or three. In wt, the first abaxial trichome appeared on average on leaf 11.4, whereas *At*GRP7ox again presented a significantly longer juvenile phase with the first abaxial trichome appearing on average on leaf 14.3. *At*GRP7ox MIM156 plants displayed an intermediate phenotype and formed the first abaxial trichome between leaves five to six. Specifically, MIM156 reduced the juvenile vegetative phase in wt plants by approximately 75–80%, whereas the reduction was only 50–60% in *At*GRP7ox MIM156 plants. Notably, the intermediate phenotype of *At*GRP7ox MIM156 plants was consistently observed across four independent experiments (Fig. 5A; Additional File 1, Fig. S8A, B and E; Additional File 6), indicating that this effect is highly robust.

Thus, the juvenile phase of *At*GRP7ox MIM156 plants was significantly shortened but not to the extent observed in MIM156 plants, pointing towards miR156-dependent and additional miR156-independent effects in *At*GRP7ox plants.

When we introgressed MIM156 into *grp7-1* and *grp7-1 grp8-2*, abaxial trichome formation was unexpectedly delayed (Fig. 5A; Additional File 6). The first abaxial trichome appeared on average on leaf 10.6 in *grp7-1* and between leaf 12 and 13 in *grp7-1* MIM156. In *grp7-1 grp8-2*, the first abaxial trichome formed on average on leaf 10.4, whereas in *grp7-1 grp8-2* MIM156, it was visible between leaf 12 to 13. This effect was also observed in a second independent experiment, where the mimicry construct had no effect on the abaxial trichome formation in the *grp7-1* and *grp7-1 grp8-2* background (Additional File 1, Fig. S8B). Although the difference between wt, *grp7-1*, and *grp7-1 grp8-2* failed to pass significance level testing, we observed a clear trend towards earlier abaxial trichome formation. This contrasts with five independent biological experiments, in which the loss-of-function lines developed their first abaxial trichome significantly earlier than wt (representative experiment in Fig. 5B), demonstrating that the phenotype is reproducible despite some experimental variability. To check for a possible dose-dependent effect, we monitored the expression of *INDUCED BY PHOSPHATE STARVATION 1* (*IPS1*), the backbone of the MIM156 construct [14, 15], and found that the construct was indeed reduced in *grp7-1* MIM156 plants and *grp7-1 grp8-2* MIM156 plants compared with the MIM156 parental line (Additional File 1, Fig. S8C).

To leverage the reduced expression of the construct in the loss-of-function backgrounds to levels comparable to those of the MIM156 parental line, we backcrossed *grp7-1* MIM156 to MIM156 (BC *grp7-1* MIM156) and confirmed restoration of expression levels in BC *grp7-1* MIM156 via RT-qPCR (Additional File 1, Fig. S8D). However, there was no effect on the length of the juvenile phase in the backcrossed population. The first abaxial trichome formed on average on leaves 10 to 11 in BC *grp7-1* MIM156 and on leaf 10.5 in *grp7-1* (Additional File 1, Fig. S8E; Additional File 6). Thus, in the absence of *At*GRP7, sequestration of miR156 did not affect the trichome phenotype.

Taken together, the introgression of MIM156 into *At*GRP7ox did not fully suppress the prolonged juvenile vegetative phase, suggesting a partially miR156-independent function of *At*GRP7 in trichome formation. MIM156 did not affect the length of the juvenile phase in the *grp7-1* and *grp7-1 grp8-2* loss-of-function mutants.

Given that *SPL3* showed the most consistent differences and shows a binding site of *At*GRP7 in the 3´UTR (Additional File 1, Fig. S9A), we tested whether the observed changes in expression were attributable to a direct effect of *At*GRP7 on *SPL3* or mediated indirectly by altered miR156 expression levels. Therefore, we measured *SPL3* expression in *At*GRP7ox MIM156, BC *grp7-1* MIM156 and MIM156. In two MIM156 lines, *SPL3* was elevated 3.5- to 5-fold compared to wt plants, indicating the loss of miR156-based downregulation. In *At*GRP7ox plants, *SPL3* was significantly reduced to approximately 50% of the wt level. In contrast, *SPL3* was not influenced in *grp7-1*. In *At*GRP7ox MIM156 and BC *grp7-1* MIM156, *SPL3* expression did not differ from that in the MIM56 parental line (Fig. 5C). These findings indicate that *SPL3* is regulated mainly through miR156 and that altered *SPL3* levels in *At*GRP7 mutants are a consequence of altered miR156-based regulation rather than being directly influenced by *At*GRP7.

Taken together, introduction of MIM156 in *At*GRP7ox partially suppressed the prolonged vegetative phase, indicating that the phenotype is dependent on miR156, while also involving miR156-independent effects. When introduced in the loss-of-function lines, MIM156 had no effect on the length of the juvenile vegetative phase. Furthermore, we identified an *At*GRP7 binding site on *SPL3*, but *SPL3* transcript levels were unchanged between *At*GRP7 mutant lines when miR156 activity was suppressed by MIM156. This indicates that *At*GRP7 affects *SPL3* expression indirectly via modulation of miR156 levels rather than through a direct regulatory interaction.

## Discussion

By applying iCLIP2 to *AtGRP7-GFP hyl1-2* plants we could systematically identify in vivo RNA-binding sites of *At*GRP7 on low-abundant pri-miRs. This points to a previously underappreciated role of *At*GRP7 in the post-transcriptional regulation of miR-related pathways. As a representative example, we investigated the miR156 pathway, which regulates the juvenile to adult vegetative phase change [1, 3, 8, 17]. We found that *At*GRP7 binds the pri-miRs *MIR156A* and *MIR156C *in vivo, affects their expression, and influences the timing of vegetative phase transition. Specifically, loss of *At*GRP7 accelerated the juvenile-to-adult transition, whereas overexpression of *At*GRP7 led to a delayed transition.

*At*GRP7ox plants displayed juvenile traits for longer, as the first abaxial trichomes appeared later, the leaf base angle was wider and the length-to-width ratio of leaf 11 was smaller. In contrast, the *grp7-1* and *grp7-1 grp8-2* mutants presented a shortened juvenile phase, with earlier trichome formation, a narrower base angle and greater length-to-width ratio on leaf 11. Loss of *At*GRP8 had no additive effect on the duration of the juvenile vegetative phase, as the duration of the vegetative phase did not differ between *grp7-1* and *grp7-1 grp8-2*. This is consistent with earlier findings showing that despite a high degree of redundancy between *At*GRP7 and *At*GRP8, the two proteins differ in their response to cold or in the downregulation of the floral repressor *FLC* [25, 26].

In addition to affecting the juvenile-to-adult vegetative phase transition, *At*GRP7 also affected overall vegetative growth dynamics. Compared to wt Col-0 plants, *At*GRP7ox plants showed a slower leaf emergence rate and generally smaller rosette size. Despite the prolonged juvenile phase, *At*GRP7ox plants exhibited a shortened adult phase and flowered earlier with fewer leaves, likely due to their reduced leaf emergence rate. In contrast, leaf emergence and appearance were not altered in *grp7-1* and *grp7-1 grp8-2* plants, but both lines transitioned to reproductive growth later than wt Col-0 plants, leading to later flowering. The additional loss of *At*GRP8 did not affect the length of the juvenile phase but further delayed flowering, indicating that *At*GRP8 is more relevant for flowering time control.

Together, these developmental phenotypes suggested that *At*GRP7 affects vegetative phase transition, prompting us to investigate miR-related mechanisms. With iCLIP2, we identified in vivo binding of *At*GRP7 to the miR156 precursors *MIR156A* and *MIR156C* and to the closely related miR157 precursors *MIR157A* and *MIR157C*. Consistent with *At*GRP7 binding, *MIR156A* and *MIR156C* transcript levels were elevated in *At*GRP7ox and reduced in *grp7-1 grp8-2* during early developmental stages. In contrast, *MIR157A* showed only a moderate increase in *At*GRP7ox beginning in week two, whereas *MIR157C* remained unchanged. Although miR156 and miR157 have overlapping functions, their relative contributions depend on developmental stage and process, with miR156 playing a more prominent role in the juvenile-to-adult vegetative transition, whereas miR157 becomes increasingly important during later development and flowering, particularly under SD conditions [7, 13, 18]. We therefore focused our functional analyses on miR156. Publicly available expression data further support a potential spatial relationship between *At*GRP7 and miR156: The AtGenExpress developmental expression data, visualized by ePlant [60], indicate strong expression of *AtGRP7* in the shoot apex and developing vegetative tissues (Additional File 1, Fig. S10), while previous studies have shown that *MIR156A* and *MIR156C* are expressed in young shoot apices and developing leaf primordia during early vegetative development [13, 59, 61]. Moreover, the changes in *MIR156A* and *MIR156C* precursor abundance were also detected in apically enriched material, providing further context for a potential *At*GRP7-miR156 regulatory relationship during the juvenile-to-adult vegetative transition.

Although *At*GRP7 binding affected the abundance of *MIR156A* and *MIR156C*, changes in mature miR156 were relatively small, suggesting *At*GRP7 may affect miR156 function beyond precursor abundance. *At*GRP7 binding may restrict access of the miRNA processing machinery to bound pri-miRNAs, consistent with previous observations that *At*GRP7 impairs pri-miRNA processing and colocalizes with DCL1 and SE in nuclear bodies [28]. Direct binding of *At*GRP7 to pri-miRNAs was proposed to interfere with processing through steric hindrance or conformational changes [28]. In addition, liquid-liquid phase separation and RNA recruitment by *At*GRP7 may influence RNA accessibility [35], although this remains untested for *MIR156A/C*. At the level of mature miRNA, altered subcellular distribution, stability or AGO1 loading could affect miR156 activity without substantially changing its total abundance. Given the reported variation in miR156 abundance between successive leaf primordia [7], spatially resolved analysis of defined primordia may help clarify these effects. Thus, we cannot exclude that *At*GRP7 also affects vegetative phase change through miR156-independent mechanisms.

This is also supported by introgression of MIM156 in the *At*GRP7ox background. *At*GRP7ox MIM156 plants presented an intermediate phenotype, with the juvenile vegetative phase being shorter than that of *At*GRP7ox plants but not as drastically reduced as that of MIM156 plants. These findings suggest that altered miR156 activity contributes to the prolonged juvenile vegetative phase of *At*GRP7ox but is unlikely to fully account for the observed phenotype. Gibberellin (GA) acts at least partly upstream of the miR156-SPL-pathway and accelerates vegetative phase change in Arabidopsis [3]. However, a GA-dependent effect in plants with altered *At*GRP7 expression has so far only been implied in terms of flowering time control [62, 63].

In contrast to its effect in the *At*GRP7ox background, MIM156 did not alter the length of the vegetative phase in the *grp7-1* or *grp7-1 grp8-2* background, and trichome formation resembled that of the parental lines. To exclude insufficient *MIM156* expression as a cause, we generated additional lines with higher *MIM156* levels, yet the phenotype remained unchanged. However, target *SPL3* levels were elevated in both the *At*GRP7ox MIM156 and the *grp7-1 grp8-2* MIM156 lines, showing that the target mimicry construct was functional and successfully sequestered miR156 (Fig. 5C; Additional File 1, Fig. S8C and D). These observations suggest that sequestration of miR156 alone is insufficient to modify vegetative phase transition in the loss-of-function backgrounds, implying that additional factors contribute to the phenotype, whereas *At*GRP7 overexpression creates conditions in which the target mimic can more effectively influence phase transition timing. Differences in cellular context or miR processing dynamics may further modulate MIM156 efficiency and underscore the context dependence of *At*GRP7–miR156 interactions.

MiR156 regulates a suite of SPL transcription factors involved in development and stress responses. SPLs can be grouped by function: *SPL2/9/10/11/13/15* contribute to both the juvenile-to-adult and vegetative-to-reproductive transitions, with *SPL9/13/15* being more important. *SPL3/4/5* also promote floral meristem identity [9, 17, 64], whereas *SPL6* is linked to defense [24].

We examined the expression of *SPL3*, *SPL9* and *SPL15*, which are known to exhibit transcriptional responses to miR156 [3, 9, 16, 64]. Consistent with the comparatively modest changes in mature miR156 abundance, transcriptional responses of the analyzed *SPL* genes were also limited. *SPL3* expression was reduced in *At*GRP7ox and slightly elevated in *grp7-1 grp8-2*, whereas *SPL9* showed only minor changes and *SPL15* remained largely unaffected. These findings suggest that the developmental effects associated with altered *At*GRP7 expression cannot be explained solely by transcriptional regulation of the canonical miR156-SPL pathway. Given that miR156 primarily regulates SPL activity post-transcriptionally, changes at the protein level or altered SPL activity cannot be excluded by transcript analyses alone [7, 64]. Furthermore, *SPL3* is reported to be among the most strongly miR156-repressed *SPL* genes [7], making it the most likely candidate to reflect subtle changes in miR156 activity. Also, we specifically considered whether the altered *SPL3* expression observed in our genotypes could provide a link between *At*GRP7, miR156 activity, and downstream regulation as we observed a binding site of *At*GRP7 on *SPL3*. Therefore, we tested whether the observed changes in expression were attributable to a direct effect of *At*GRP7 on *SPL3* or dependent on miR156 expression levels by introgression of the MIM156 construct. As *SPL3* levels in the MIM156 introgression lines were unchanged compared to those in the MIM156 parental line, we suggest that the observed *SPL3* differences are primarily explained by altered miR156 abundance rather than by direct regulation of *SPL3* through *At*GRP7. Thus, *At*GRP7 likely influences vegetative phase change through mechanisms beyond transcriptional regulation of the canonical miR156-SPL pathway. To further explore potential direct targets beyond the canonical miR156-SPL pathway, we screened genes implicated in vegetative phase change (recently reviewed in [65]) for *At*GRP7 binding sites. Binding was detected on *TOE1*, *TCP4* and *BIN2*, while *SPL3* was the only miR156-regulated *SPL* gene with a binding site (Additional File 1, Fig. S9A-F). Thus, *At*GRP7 may directly interact with additional regulators of vegetative phase change, although the functional consequences of these interactions remain to be determined.

These findings further emphasize the importance of understanding the broader repertoire of *At*GRP7-bound pri-miRs. Therefore, we applied iCLIP2 in the *hyl1-2* mutant background, which is impaired in miR processing and accumulates pri-miRs [45, 46]. In these plants, *At*GRP7 binding sites on 44 pri-miRs were detected. Consistent with previous studies [30, 31], *At*GRP7 binding sites identified by iCLIP2 were enriched for a characteristic uracil-rich motif flanked by guanine and cytosine, supporting the robustness of our dataset. The binding sites were distributed over the entire length of the pri-miRs, showing no clear preference for binding positions within the transcripts. Consistent with its known RNA-binding properties [57], *At*GRP7 binding sites predominantly associated with less structured, single-stranded regions. We further examined the spatial relationship between *At*GRP7 binding sites located within miRNA hairpins and experimentally determined DCL1 cleavage sites [58]. In several cases, *At*GRP7 binding sites were located close to a DCL1 cleavage site, with *MIR156C*, *MIR160C*, *MIR168A* and *MIR159B* showing binding sites within 11–26 nt of at least one cleavage site, whereas the *MIR393B* binding site was located further downstream (Fig. 3G, Additional File 1, Fig. S6A-E). Thus, several *At*GRP7 binding sites occur in proximity to regions involved in pri-miRNA processing, although no single defined positional relationship was apparent. The comparable nucleotide composition across all binding sites, including *MIR*-associated and unambiguously bound *MIRs*, indicates that *At*GRP7 recognizes similar sequence features independent of transcript class.

Compared to our earlier iCLIP1 dataset [31], the iCLIP2 protocol and the use of the *hyl1-2* background markedly increased the sensitivity allowing for more stringent filtering and robust identification of bona fide binding sites, enabling the detection of multiple binding sites on transcripts that previously showed only weak or no signals. On average we recovered tenfold more crosslink sites on *MIRs* compared to the iCLIP1 dataset [31]. Apart from binding to *MIR156A* and *MIR156C*, other bound pri-miRs are involved in the regulation of diverse processes, underscoring the broad functional relevance of *At*GRP7–miR interactions.

Taken together, our optimized iCLIP2 pipeline using the *hyl1-2* background provides a robust method for identifying *At*GRP7 binding sites on low abundant pri-miRs and reveals the broad contribution of *At*GRP7 to post-transcriptional regulation of miR-related pathways.

## Conclusion

Our findings demonstrate that *At*GRP7 directly associates with multiple pri-miRs in vivo, including *MIR156A* and *MIR156C*, identified through our iCLIP2 approach. Perturbation of *At*GRP7 expression affects the timing of vegetative phase transition, as *At*GRP7 overexpression prolongs the juvenile phase while loss-of-function shortens it. While altered miR156 activity contributes to this phenotype, the relatively small changes in mature miR156 levels, limited effects on *SPL* abundance, and partial rescue by MIM156 indicate that *At*GRP7-dependent regulation of vegetative development cannot be explained solely by the canonical miR156-SPL pathway. Instead, our data suggest that *At*GRP7 integrates miR-related regulation with additional post-transcriptional mechanisms to control developmental transitions. Together, our findings establish *At*GRP7 as an RNA-binding protein that interacts with a broad set of pri-miRs in vivo and highlight how RNA-binding proteins may fine-tune developmental regulation through complex post-transcriptional networks.

## Supplementary Material

Below is the link to the electronic supplementary material.

Additional File 1

Additional File 2

Additional File 3

Additional File 4

Additional File 5

Additional File 6

Additional File 7

## Data Availability

The data reported here are deposited in the Gene Expression Omnibus database under accession numbers SRR36909813, SRR36909812 & SRR36909811 (iCLIP2). Data from [31] can be found under accession number GSE99427 (iCLIP1). 35S::GFP iCLIP2 data from [51] can be found in the European Nucleotide Archive under accession number PRJEB71752.
