# Supplementary material for: MiR156-dependent and miR156-independent effects of the RNA-binding protein AtGRP7 on vegetative phase change in Arabidopsis
Source: BMC Plant Biol. 2026 Oct 17;26:1951. doi: 10.1186/s12870-026-10084-w (PMC13761024; doi:10.1186/s12870-026-10084-w)
Supplement: Supplementary file 1 [file 12870_2026_10084_MOESM1_ESM.pdf]

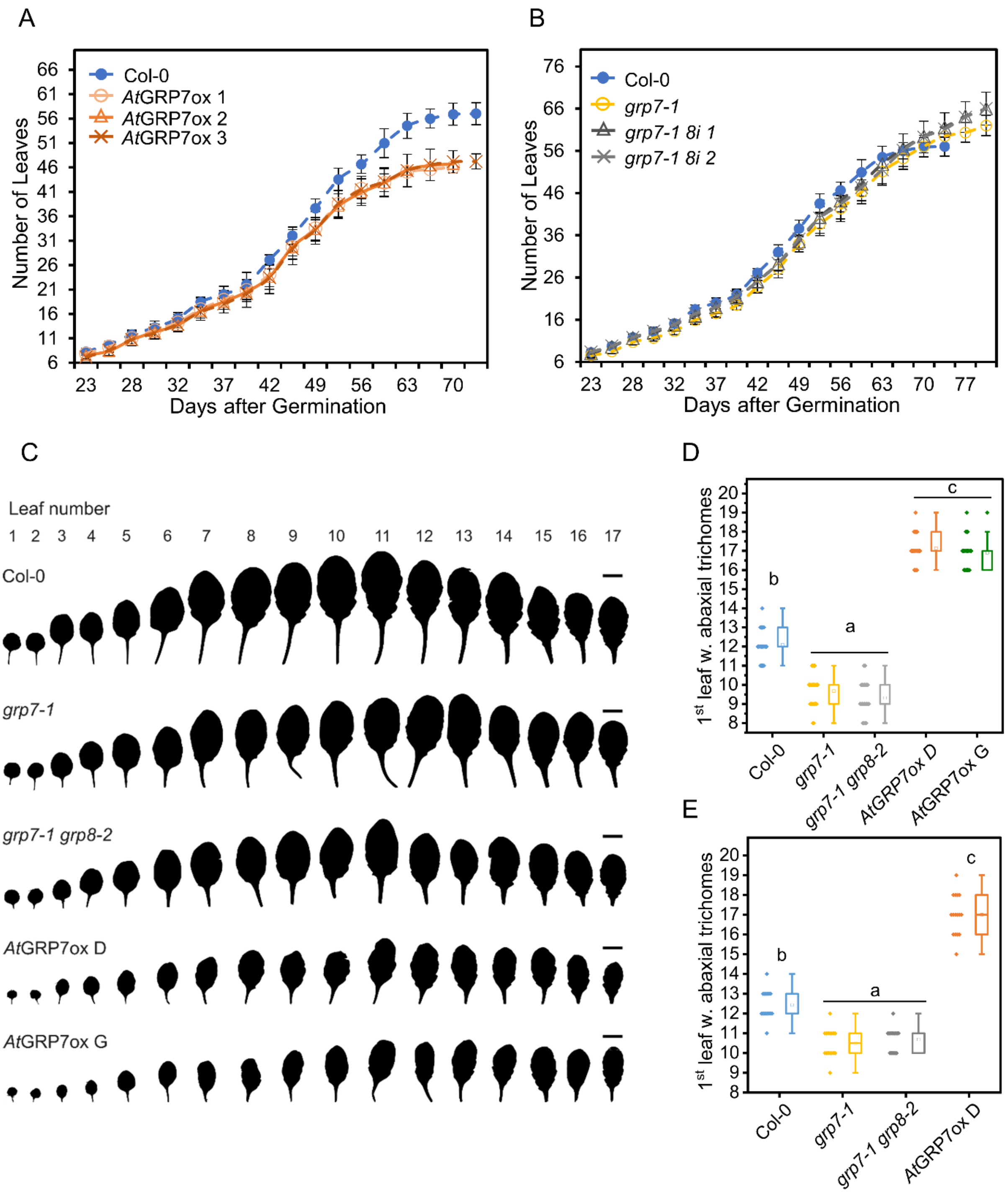

Supplementary Figure 1: Additional data supporting the vegetative phenotype of *At*GRP7 mutants.

Leaf emergence of **(A)** Col-0 and three independent *At*GRP7 overexpressing lines, and **(B)** Col-0, *grp7-1*, and two double loss-of-function lines. All visible leaves were counted three times per week until flowering. Data are presented as mean ± standard deviation. **(C)** Fully expanded rosette leaves of 45-day-old wt Col-0, *grp7-1*, *grp7-1 grp8-2*, and two *At*GRP7ox lines grown under SD conditions. Scale bar = 1 cm. **(D)** Leaf number at the appearance of the first abaxial trichome in wt Col-0, *grp7-1*, *grp7-1 grp8-2*, and two *At*GRP7ox lines (n = 21–25). **(E)** Leaf number at the appearance of the first abaxial trichome in wt Col-0, *grp7-1*, *grp7-1 grp8-2*, and *At*GRP7ox (n = 16). Data in (D) and (E) are shown as box-and-whisker plots (median, mean, 25th–75th percentiles, and 5th–95th percentiles) with individual data points overlaid. Different letters indicate significant differences (Tukey's HSD, $p < 0.05$). Plants were grown under SD conditions

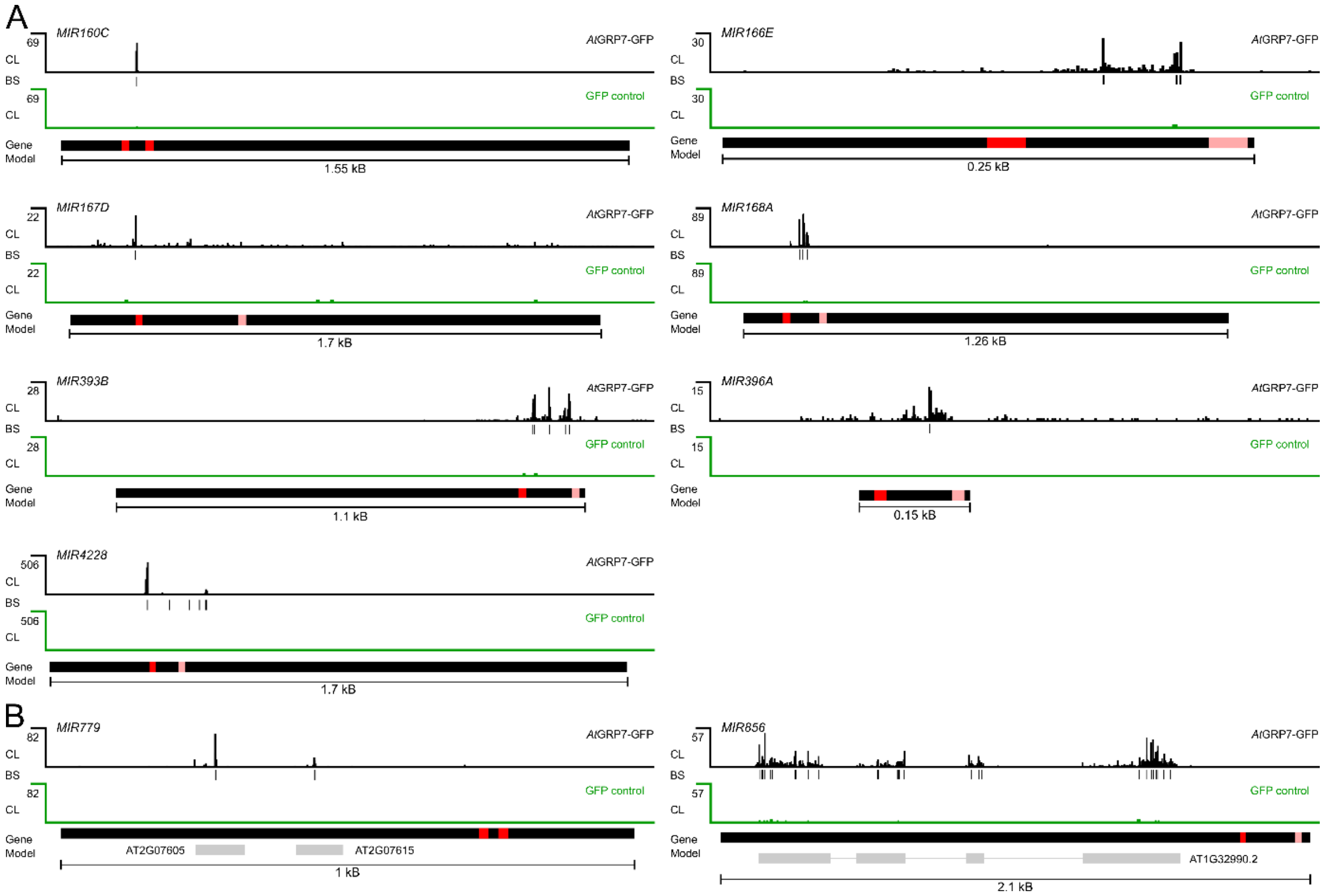

Supplementary Figure 2: Pri-miRs bound by *At*GRP7 identified in previous iCLIP1.

(A) Unambiguously bound pri-miRs and **(B)** pri-miRs with binding sites likely derived from overlapping genes based on the iCLIP-data from [31]. For each locus, crosslink (CL) tracks and called binding sites (BS) from the *AtGRP7–GFP grp7-1* sample (black) and the 35S::GFP control (green) are shown. Gene model of the highest expressed pri-miR isoform (based on RNA-seq data from [31]; annotated to AtRTD3) is shown in 5′→3′ orientation (exons: boxes; introns: lines). Mature miR and miR* positions depicted in red; predicted miR* sites in apricot. Overlapping genes are shown in gray. The size of the displayed region is indicated.

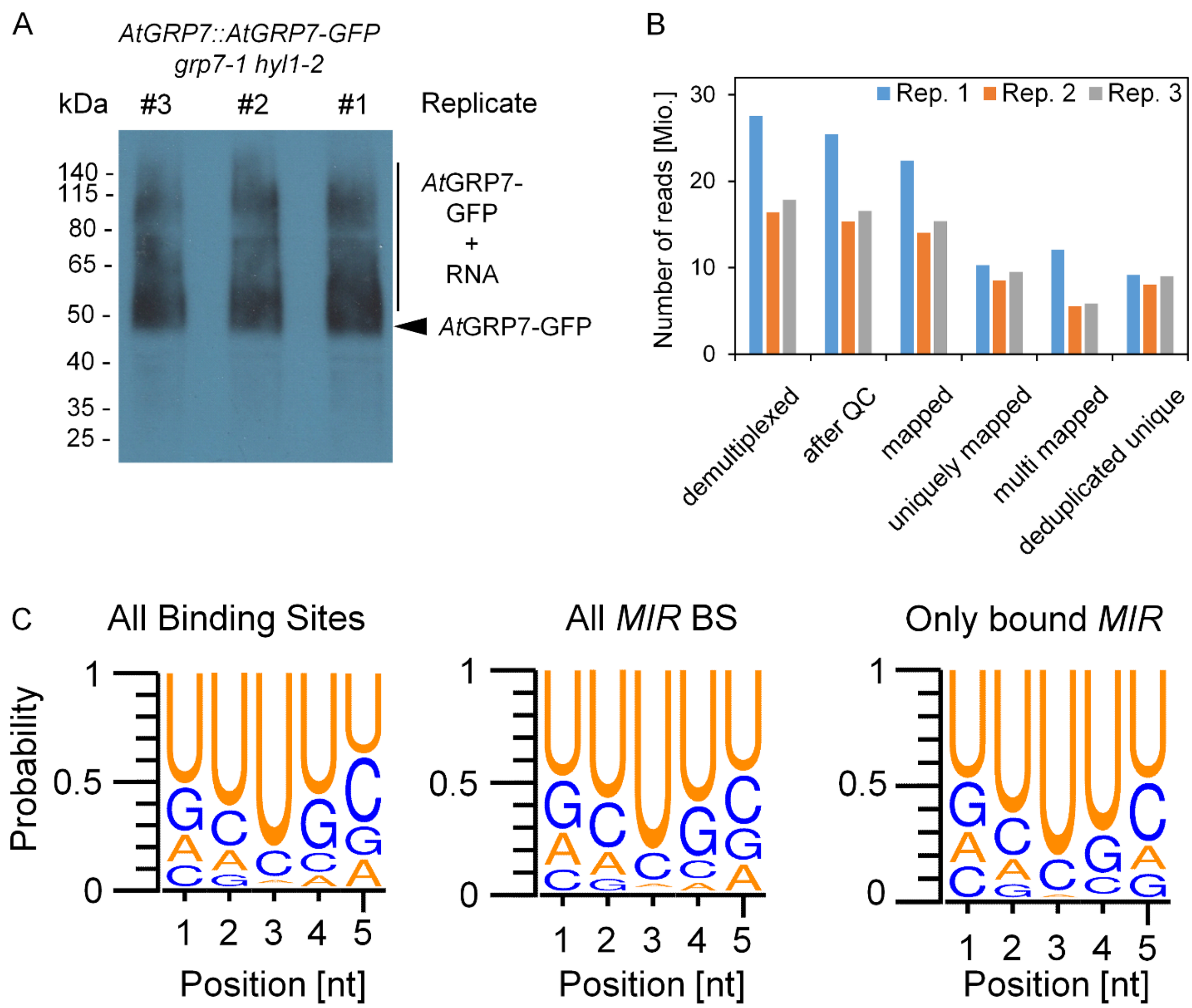

Supplementary Figure 3: Statistics of iCLIP2 using *AtGRP7::AtGRP7-GFP grp7-1 hyl1-2*.

(A) Autoradiogram of RNA–protein complexes from three independent replicates of UV-crosslinked *AtGRP7::AtGRP7-GFP grp7-1 hyl1-2* plants after immunoprecipitation with α-GFP Trap beads. Positions of the molecular weight markers are indicated. The autoradiogram was exposed for 2 hours. The uncropped radiogram can be found in Additional File 3. **(B)** Sequencing statistics for each replicate, showing each step of the bioinformatics pipeline: demultiplexing, quality control, and mapping against the AtRTD3 reference. Proportions of multi-mapped and uniquely mapped reads, as well as deduplicated uniquely mapped reads, are shown, which served as the basis for further analysis. **(C)** Proportion of each nucleotide at each position of the 5 nt-long binding sites identified by iCLIP2. *All MIR binding sites* are all binding sites at genes identified as *MIR*. *Only bound MIR* depicts all binding sites at unambiguously identified *MIRs*. Abbreviations: BS; binding site.

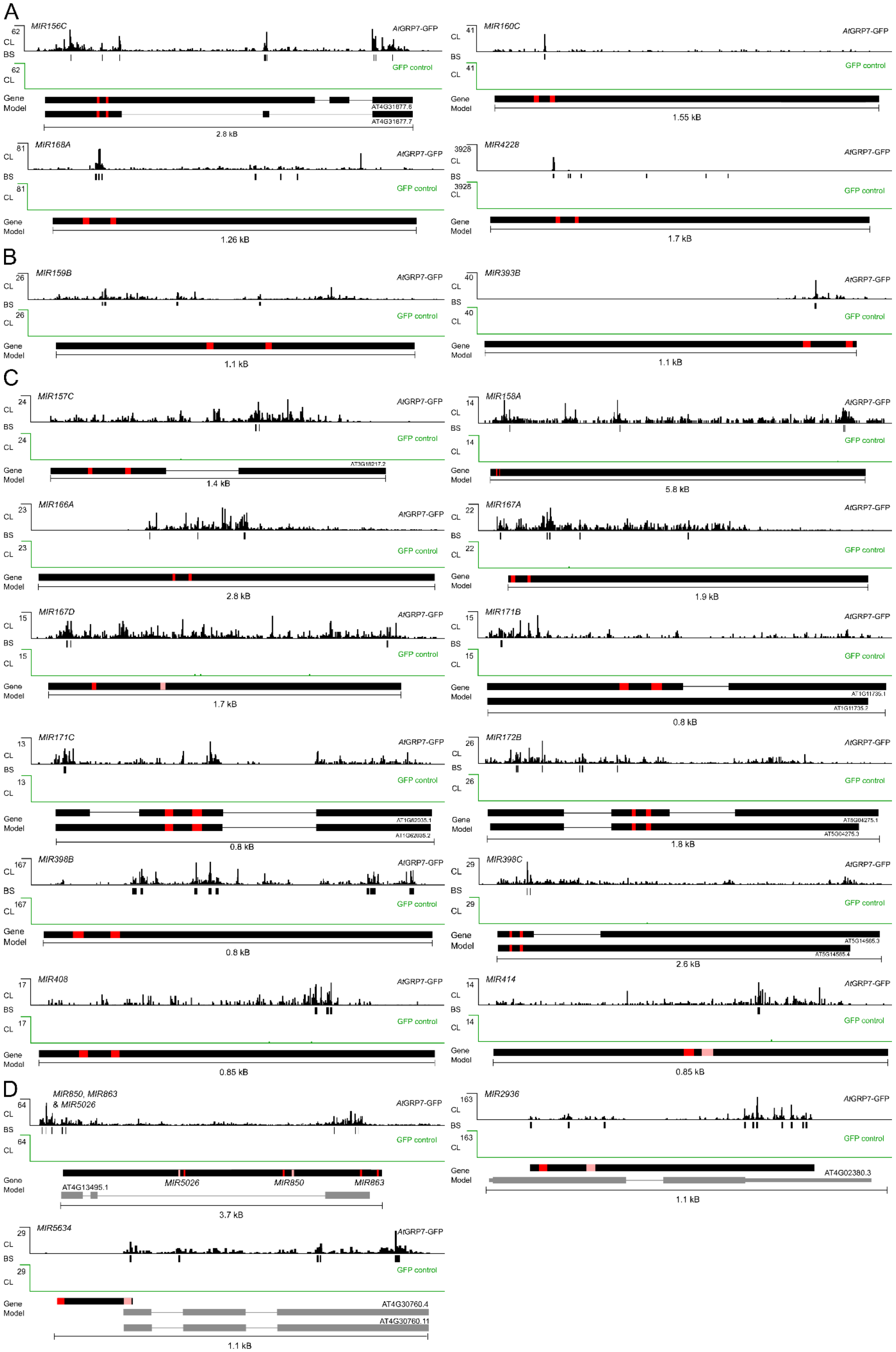

Supplementary Figure 4: Pri-miRs bound by *At*GRP7 identified by iCLIP2.

(A) Loop-bound, **(B)** hairpin-bound, **(C)** other bound pri-miRs, and **(D)** pri-miRs with binding sites likely derived from overlapping genes. For each locus, crosslink (CL) tracks and called binding sites (BS) from the *AtGRP7::AtGRP7–GFP grp7-1 hyl1-2* sample (black) and the 35S::GFP control (green) are shown. Gene model of the highest expressed pri-miR isoform (based on RNA-seq data from [31]; annotated to AtRTD3) is shown in 5′→3′ orientation (exons: boxes; introns: lines). Mature miR and miR* positions depicted in red; predicted miR* sites in apricot. Overlapping genes are shown in gray. The size of the displayed region is indicated.

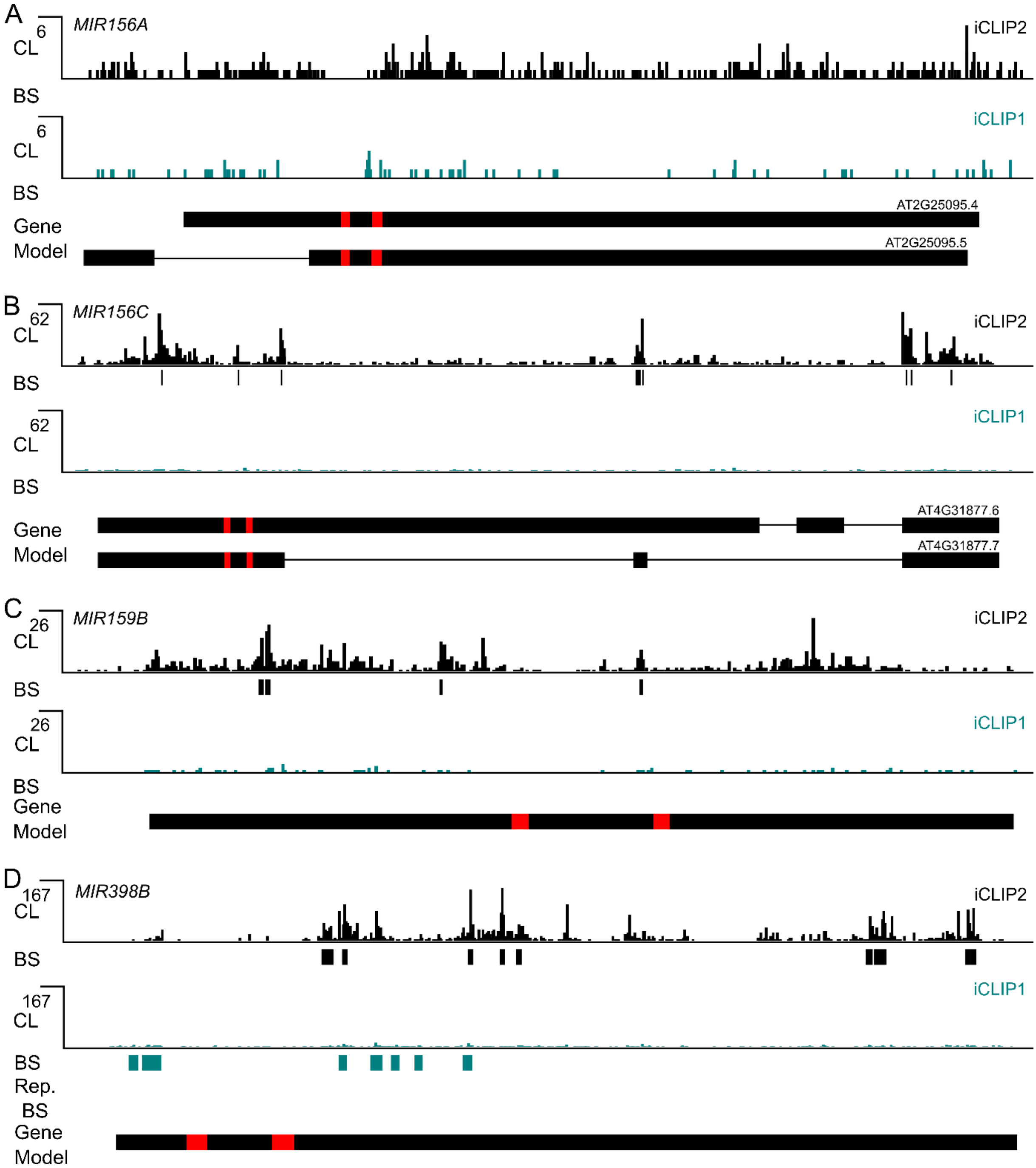

Supplementary Figure 5: Comparison of iCLIP1 with iCLIP2.

Comparison of iCLIP2 (*AtGRP7::AtGRP7-GFP grp7-1 hyl1-2*, black) and iCLIP1 (*AtGRP7::AtGRP7-GFP grp7-1*, teal, from [31]) on **(A)** *MIR156A*, **(B)** *MIR156C*, **(C)** *MIR159B*, and **(D)** *MIR398B*. For each pri-miR, crosslink tracks (CL) and called binding sites (BS) are shown. The gene model of the highest expressed pri-miR isoform (RNA-seq data from [31] annotated according to AtRTD3) is shown in 5′→3′ orientation (exons: boxes; introns: lines). Positions of mature miR and miR* are indicated in red. Representative binding sites (Rep. BS), defined as sites appearing in at least two of three biological replicates, are highlighted. This filtering step is integrated into the bioinformatic pipeline of the iCLIP2 protocol.

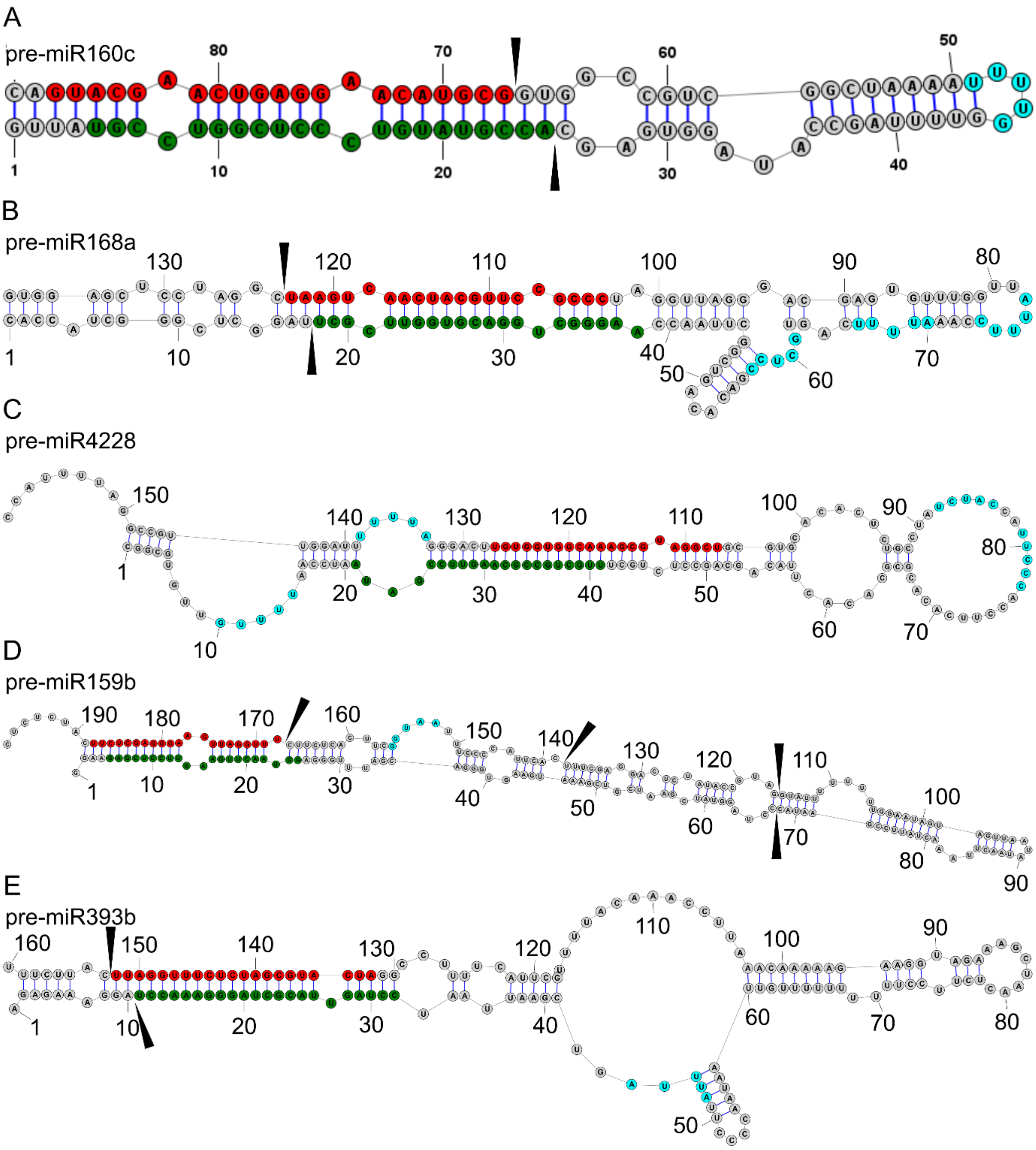

Supplementary Figure 6: *At*GRP7 binds preferentially single-stranded regions.

Secondary structures of **(A)** *pre-miR160c*, **(B)** *pre-miR168a*, **(C)** *pre-miR4228*, **(D)** *pre-miR159b*, and **(E)** *pre-miR393b* predicted by RNAFold. Highlighted are 5p-miR (green), 3p-miR (red) as well as *At*GRP7-GFP binding sites (cyan) determined by iCLIP2 in *AtGRP7::AtGRP7-GFP grp7-1 hyl1-2*. Experimentally determined DCL1 cleavage sites [58] are indicated by black arrows.

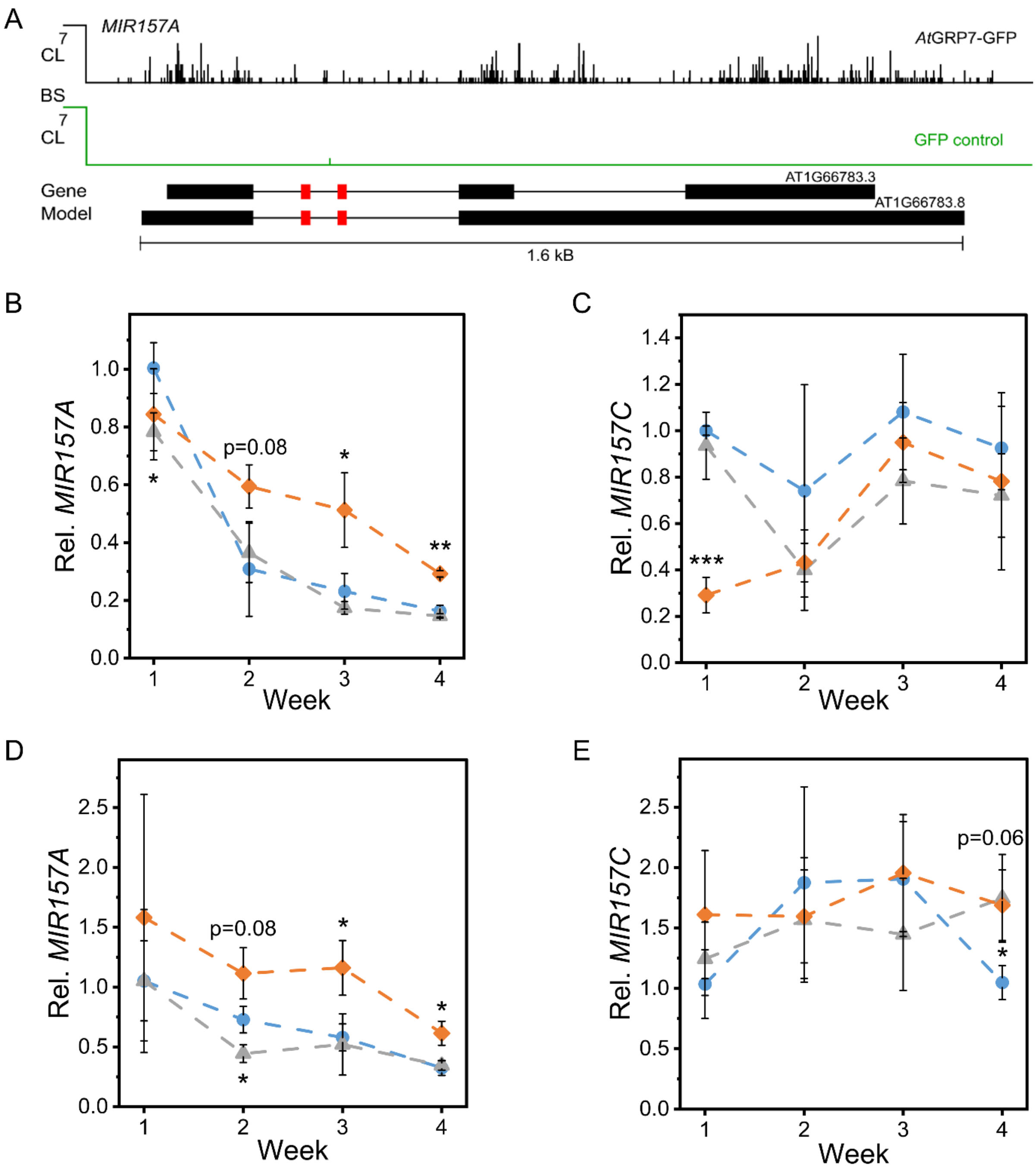

Supplementary Figure 7: Influence of *At*GRP7 on miR157-precursors *MIR157A* and *MIR157C*.

(A) *At*GRP7 iCLIP2 binding profiles on *MIR157A*. Crosslink (CL) tracks from the *At*GRP7::*At*GRP7–GFP *grp7-1 hyl1-2* (black) and GFP control (green) are shown. Called binding sites (BS) are marked by black lines. Gene models of the highest expressed pri-miRNA isoforms (based on RNA-seq data from [31]; annotated according to AtRTD3) are shown from left to right in 5´→3´ orientation, with exons (boxes), introns (lines), and the miR/miR* regions highlighted in red. The size of the displayed region is indicated. Levels of **(B, D)** *MIR157A* and **(C, E)** *MIR157C* in Col-0, *grp7-1 grp8-2*, and *At*GRP7ox plants. Whole rosettes (B and C) or apically enriched tissue (D and E) from 1- to 4-week-old plants grown under SD conditions were analyzed via RT-qPCR. Expression values were calculated using the $\Delta\Delta C_t$ method, normalized to *PP2A*, and set relative to Col-0 at week 1. The data represent means ± SD of three biological replicates. Statistical significance compared with Col-0 was assessed by Student's t-test (*** $p \leq 0.001$; ** $p \leq 0.01$; * $p \leq 0.05$; n.s. $p \geq 0.05$).

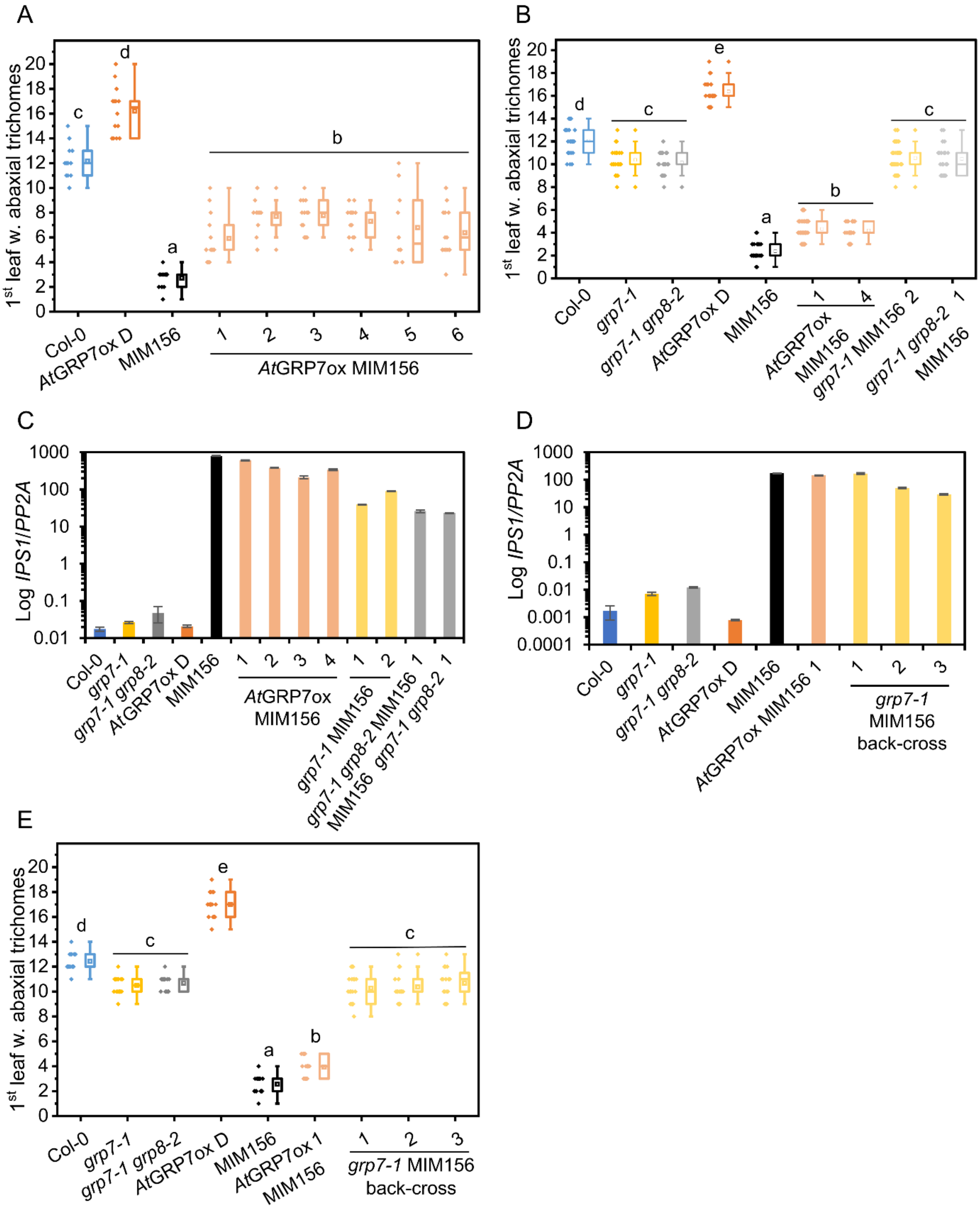

Supplementary Figure 8: Additional MIM156 data.

Leaf number of the first abaxial trichome in **(A)** Col-0, *At*GRP7ox, MIM156 lines and their crosses (n = 14), as well as in **(B)** Col-0, *grp7-1*, *grp7-1 grp8-2*, *At*GRP7ox, MIM156, and their crosses (n = 16). **(C, D)** *IPS1* expression of the corresponding plants, measured by RT-qPCR and normalized to *PP2A* (mean ± SD of two technical replicates; y-axis log-scaled). **(E)** Col-0, *grp7-1*, *grp7-1 grp8-2*, *At*GRP7ox, MIM156, and backcrosses (n = 14-16). Data in (A), (B), and (E) are shown as box-and-whisker plots (median, mean, 25th–75th percentiles, and 5th–95th percentiles) with individual data points overlaid. Different letters indicate significant differences (Tukey's HSD, $p < 0.05$). Plants were grown under SD conditions.

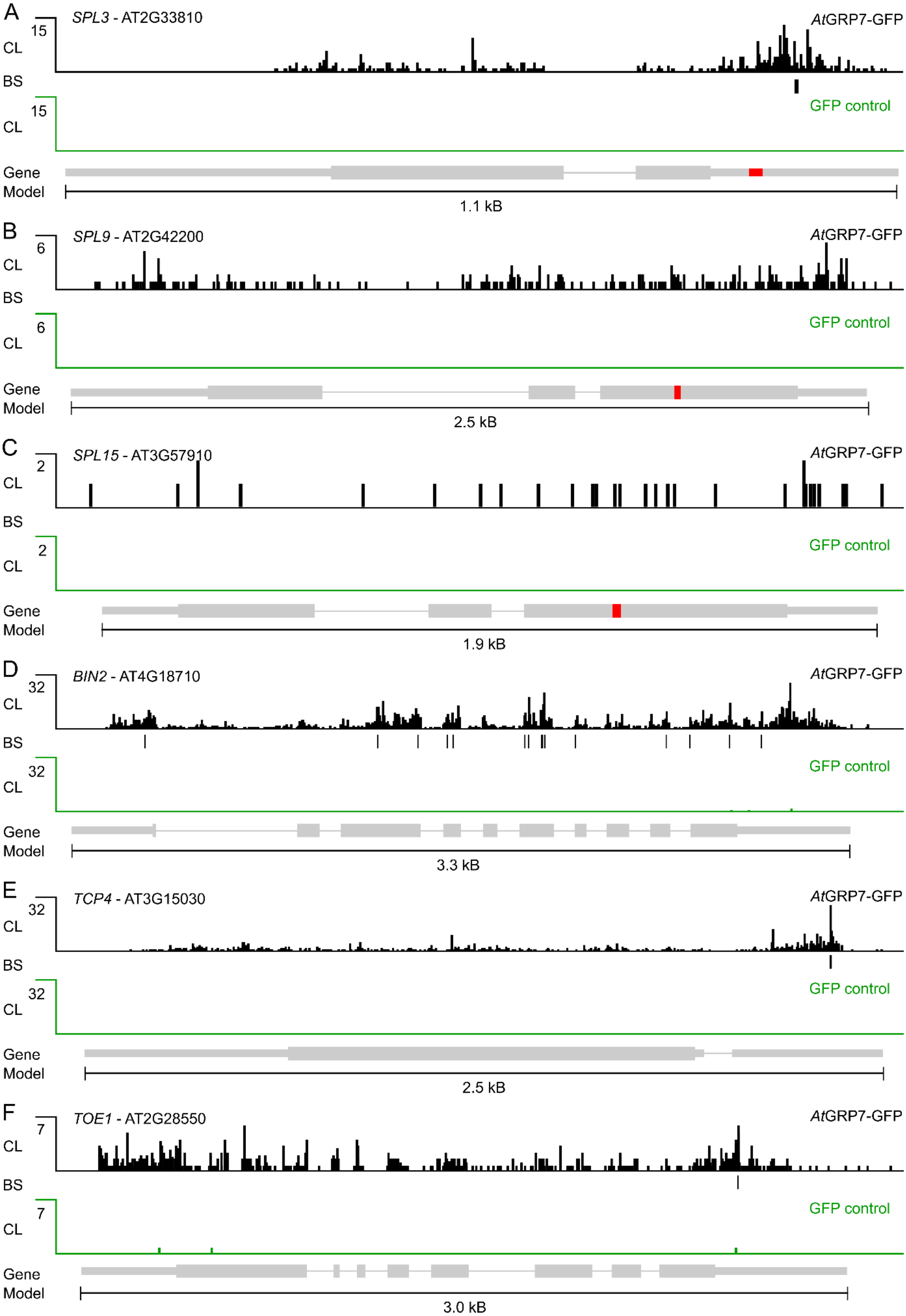

Supplementary Figure 9: *In vivo* binding sites of *At*GRP7 on genes involved in vegetative phase change.

*At*GRP7-GFP binding sites at **(A)** *SPL3*, **(B)** *SPL9*, **(C)** *SPL15*, **(D)** *BIN2*, **(E)** *TCP4*, and **(F)** *TOE1*. Crosslink tracks (CL) from the *AtGRP7::AtGRP7-GFP grp7-1 hyl1-2* (black) and the GFP (green) sample are shown. Called binding sites (BS) for *At*GRP7-GFP are indicated by black boxes. The representative gene model according to Araport11 annotation is shown below in grey (lines represent introns, boxes exons, thinner boxes UTR´s) in 5´→3´ orientation. The miR156 target site (MRE) is highlighted in red. The size of the shown region is indicated. SPL, SQUAMOSA PROMOTER BINDING PROTEIN-LIKE; BIN2, BRASSINOSTEROID INSENSITIVE2; TCP4, TEOSINTE BRANCHED1/CYCLOIDEA/PCF4; TOE1, TARGET OF EAT1.

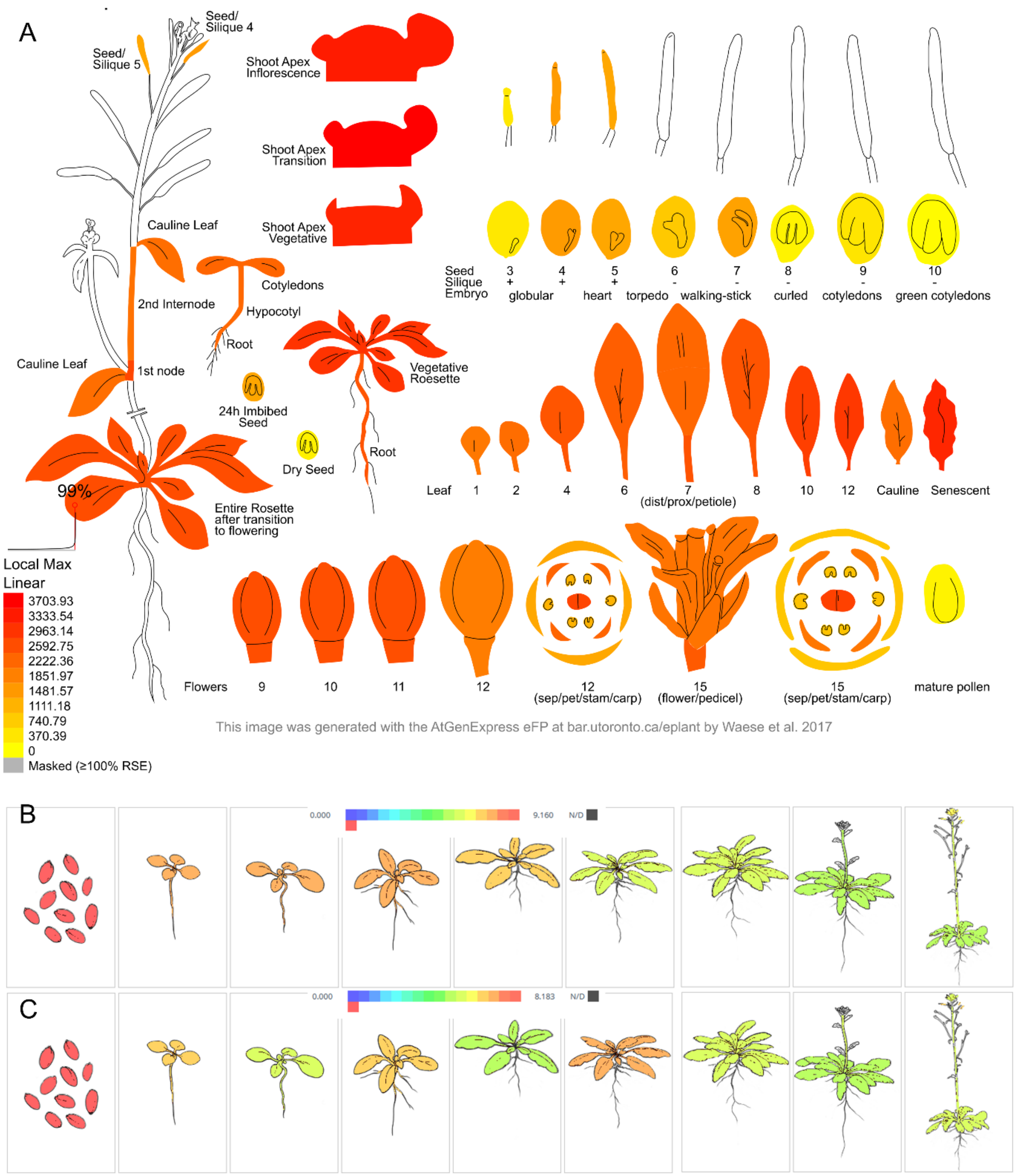

Supplementary Figure 10: Expression profiles of *AtGRP7* and *MIR156A/C* in different tissues and developmental stages.

(A) Representative screenshot of the ePlant [60] visualization of AtGenExpress eFP data for *AtGRP7*. Representative screenshots of transcript levels of **(B)** *MIR156A* and **(C)** *MIR156C* from mirEX2 [53].
