# Supplementary material for: MiR156-dependent and miR156-independent effects of the RNA-binding protein AtGRP7 on vegetative phase change in Arabidopsis
Source: BMC Plant Biol. 2026 Oct 17;26:1951. doi: 10.1186/s12870-026-10084-w (PMC13761024; doi:10.1186/s12870-026-10084-w)
Supplement: Supplementary file 2 [file 12870_2026_10084_MOESM2_ESM.pdf]

Additional File 2: Uncropped autoradiogram corresponding to Additional File 1, Fig. S3A.

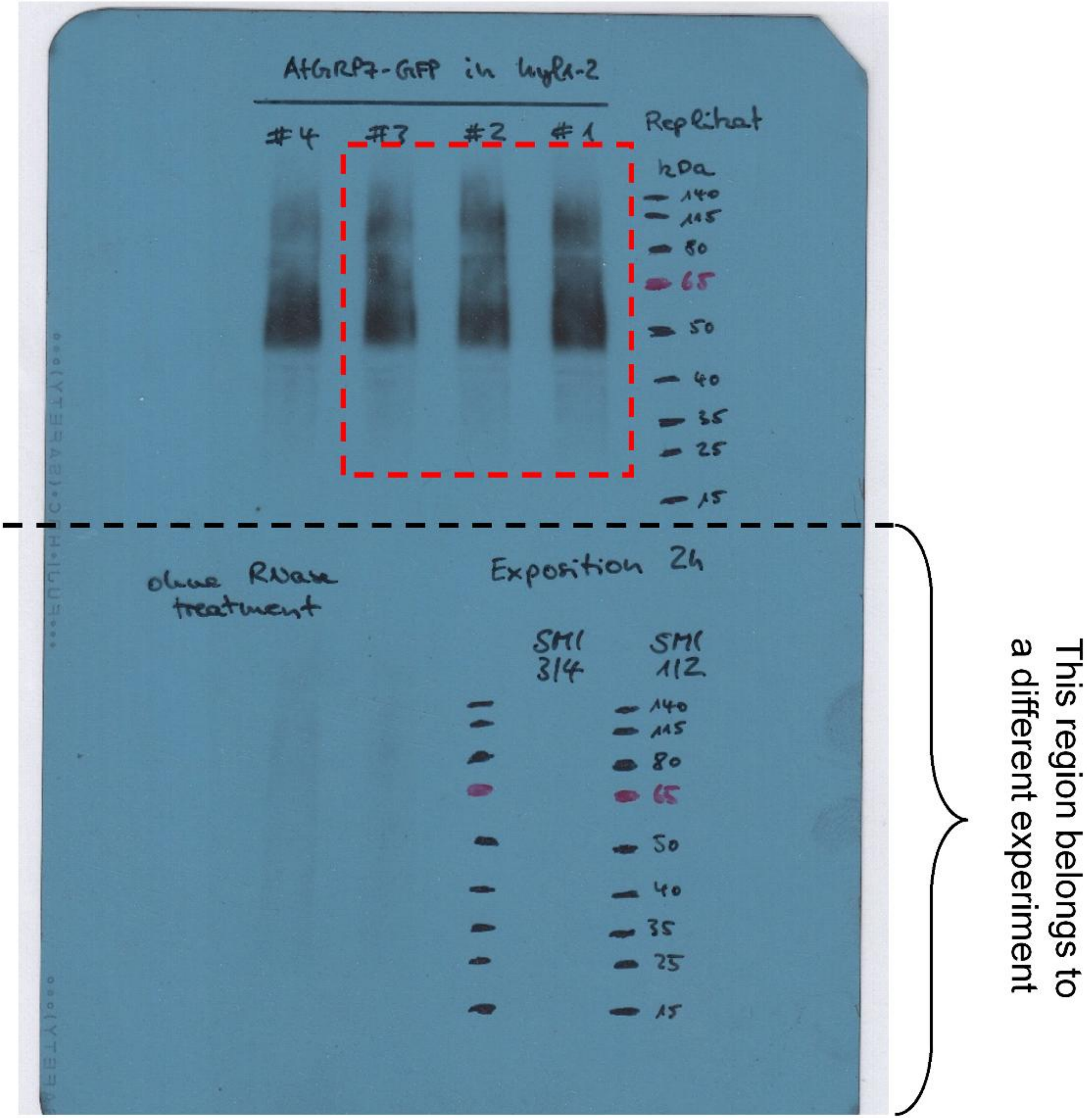

Additional File 2: Uncropped autoradiogram corresponding to Additional File 1, Figure S3A. Autoradiogram of RNA–protein complexes from four independent replicates of UV-crosslinked *AtGRP7::AtGRP7-GFP grp7-1 hyl1-2* plants after immunoprecipitation with α-GFP Trap beads. Libraries were generated from replicates 1-3, replicate 4 was not used further. Positions of the molecular weight markers are indicated. The autoradiogram was exposed for 2 hours. Dotted red box indicates the region used for Additional File 1, Fig. S3A. The region below the dotted black line belongs to a different experiment.
